# Specialization of plant–pollinator interactions increases with temperature at Mt. Kilimanjaro

**DOI:** 10.1002/ece3.6056

**Published:** 2020-02-05

**Authors:** Alice Classen, Connal D. Eardley, Andreas Hemp, Marcell K. Peters, Ralph S. Peters, Axel Ssymank, Ingolf Steffan‐Dewenter

**Affiliations:** ^1^ Department of Animal Ecology and Tropical Biology Biocenter University of Würzburg Würzburg Germany; ^2^ Unit of Environmental Sciences and Management North West University Potchefstroom South Africa; ^3^ Department of Plant Systematics University of Bayreuth Bayreuth Germany; ^4^ Department Arthropoda Zoological Research Museum Alexander Koenig Bonn Germany; ^5^ Bundesamt für Naturschutz Bonn Germany

**Keywords:** altitudinal gradient, climate change, ecological network, functional traits, generalization, mutualistic interactions, network specialization index (*H*_2_′), pollination, robustness, specialization

## Abstract

**Aim:**

Species differ in their degree of specialization when interacting with other species, with significant consequences for the function and robustness of ecosystems. In order to better estimate such consequences, we need to improve our understanding of the spatial patterns and drivers of specialization in interaction networks.

**Methods:**

Here, we used the extensive environmental gradient of Mt. Kilimanjaro (Tanzania, East Africa) to study patterns and drivers of specialization, and robustness of plant–pollinator interactions against simulated species extinction with standardized sampling methods. We studied specialization, network robustness and other network indices of 67 quantitative plant–pollinator networks consisting of 268 observational hours and 4,380 plant–pollinator interactions along a 3.4 km elevational gradient. Using path analysis, we tested whether resource availability, pollinator richness, visitation rates, temperature, and/or area explain average specialization in pollinator communities. We further linked pollinator specialization to different pollinator taxa, and species traits, that is, proboscis length, body size, and species elevational ranges.

**Results:**

We found that specialization decreased with increasing elevation at different levels of biological organization. Among all variables, mean annual temperature was the best predictor of average specialization in pollinator communities. Specialization differed between pollinator taxa, but was not related to pollinator traits. Network robustness against simulated species extinctions of both plants and pollinators was lowest in the most specialized interaction networks, that is, in the lowlands.

**Conclusions:**

Our study uncovers patterns in plant–pollinator specialization along elevational gradients. Mean annual temperature was closely linked to pollinator specialization. Energetic constraints, caused by short activity timeframes in cold highlands, may force ectothermic species to broaden their dietary spectrum. Alternatively or in addition, accelerated evolutionary rates might facilitate the establishment of specialization under warm climates. Despite the mechanisms behind the patterns have yet to be fully resolved, our data suggest that temperature shifts in the course of climate change may destabilize pollination networks by affecting network architecture.

## Graphical abstract

We studied specialization patterns in plant–pollinator interactions along a 3.4 km elevational gradient on Mt. Kilimanjaro (Tanzania) and demonstrated that specialization decreased with elevation across networks, communities, and pollinator species. Temperature predicted the degree of specialization better than pollinator richness, resource availability, area, or pollinator traits. Despite the mechanisms behind the patterns have yet to be fully resolved, we conclude that temperature shifts in the course of climate change may destabilize pollination networks by affecting network architecture.

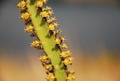

## 1 INTRODUCTION

Interspecific species interactions are known to limit the diversity and distribution of species (Bairey, Kelsic, & Kishony, 2016; Bascompte, 2006; Bastolla et al., 2009; Chan, Shih, Chang, Shen, & Chen, 2019; Wisz et al., 2013), to promote species evolution (Ramos & Schiestl, 2019), and to determine ecosystem functions (Brosi & Briggs, 2013; Garibaldi et al., 2013). Much progress was made in understanding the structure and dynamics of species interaction networks (Bastolla et al., 2009; Blüthgen, Menzel, & Blüthgen, 2006; Olesen, Bascompte, Dupont, & Jordano, 2007; Petanidou, Kallimanis, Tzanopoulos, Sgardelis, & Pantis, 2008). Nevertheless, knowledge about the spatial patterns and drivers of network properties remains surprisingly ambiguous (Morris, Gripenberg, Lewis, & Roslin, 2014; Novotny, 2006; Ollerton & Cranmer, 2002; Schleuning et al., 2012; Song, Rohr, & Saavedra, 2017; Lara‐Romero et al., 2019). Elevational gradients offer the opportunity to study the architecture of species interaction networks along broad climatic gradients at feasible spatial and temporal scales (Hoiss, Krauss, & Steffan‐Dewenter, 2015; Ramos‐Jiliberto et al., 2010; Lara‐Romero et al., 2019). Importantly, on mountains, network metrics, and potential underlying drivers can be measured in a standardized and thus informative manner. This rare information is essential for understanding the evolution and coexistence of species communities and for predicting the functionality of ecosystems under global change (Tylianakis, Laliberté, Nielsen, & Bascompte, 2010).

Plant–pollinator interactions belong to the most frequently studied mutualistic interactions in terrestrial ecosystems (Waser & Ollerton, 2006). The networks share typical topological features, such as high degrees of nestedness, arising from the tendency of specialists to interact with generalists, which tend to interact among each other (Bascompte & Jordano, 2007). Also skewed distributions of links per species resulting from the dominance of few generalists among plenty of species that only interact occasionally (Jordano, Bascompte, & Olesen, 2002), and dependence asymmetry, that is, the differences in mutual dependencies of interacting species (Bascompte, 2006; Blüthgen, Menzel, Hovestadt, Fiala, & Blüthgen, 2007), are network commonalities.

Due to its impact on pollination success and network stability, an interesting feature of plant–pollinator interactions is the species and network specialization. From a plant's perspective, higher specialization on specific pollinators may promote reproductive success and increase genetic diversity, because better morphological adaptations between the pollinator and the reproductive parts of the plant can increase the amount of transferred pollen (Waser & Ollerton, 2006). In contrast, higher generalization may decrease the dependence on specific pollen vectors and stabilize pollination over broad temporal and spatial scales (Brosi, 2016). From a pollinator's perspective, specialization is a way to reduce interspecific competition and foraging costs, as switching between different search images and handling a diverse range of flower types can be costly (Chittka & Thomson, 1997). On the other hand, specialized pollinators risk investing energy in additional flight time and ignoring lucrative floral resources nearby, which may outweigh the benefits of specialization in energy‐restricted habitats and destabilize food safety in environments with high spatial and temporal resource turnover (Waser & Ollerton, 2006). Furthermore, the adaptive potential of specialists to optimize foraging dependent on macronutrient requirements (Vaudo, Patch, Mortensen, Tooker, & Grozinger, 2016) is limited. These trade‐offs for plants and pollinators point either toward a strong selection pressure on the degree of floral specialization or, alternatively, require a high plasticity to allow for adaptive foraging, depending on the ecological context (Miller‐Struttmann & Galen, 2014; Spiesman & Gratton, 2016).

Factors that may shape the specialization of pollinator communities are, *inter alia*, the abundance and richness of floral resources, the interaction strengths among pollinators, climatic variables, and the area of available habitat. First, the abundance of floral resources determines average foraging distances and the net energy gain of pollinators (Carvell et al., 2012). Under the assumption that the net energy gain of foraging flights on average decreases with decreasing abundances of flowering plants, specialization may be favored in habitats with high abundances of flowering plants (Kunin & Iwasa, 1996). Flower richness, in contrast, sets the limits for resource partitioning. Specialization should be more likely to occur in habitats offering a variety of resources. Second, a temporarily reduced number of interactions with an otherwise common pollinator species has been shown to broaden the food choice of another pollinator (Brosi & Briggs, 2013). Frequent interspecific interactions, resulting from, for example, high pollinator richness or high visitation rates, may permanently restrict diet breadth promoting species coexistence in the long term (Goulson, Lye, & Darvill, 2008). Third, climate shapes plant and pollinator richness, composition and phenology and has been directly linked to network properties, including specialization (Petanidou et al., 2018). Temperature determines the costs of foraging flights in ectothermic pollinators (Kovac, Stabentheiner, & Brodschneider, 2015) and may thus modulate resource usage strategies in a way that species broaden their dietary spectrum in energy‐limited habitats (Miller‐Struttmann & Galen, 2014). Restricted foraging times due to persistent mist and/or temperatures below a threshold in which foraging is possible, should equally result in more generalized foraging. Furthermore, true specialization might establish faster and more often under warm climates, as evolutionary rates accelerate with temperature (Allen et al., 2006; Lin et al., 2019). Finally, habitat area may influence the mean degree of specialization in mutualistic networks (Sugiura, 2010). Specialists typically depend on larger habitat areas than generalists (Bommarco et al., 2010) and might thus become less abundant in high elevations, were habitat area is significantly reduced. Yet, the relative importance of resources, the interactions among pollinators, climate, and area in structuring plant–pollinator interactions remains unclear.

The search for factors that explain specialization is aggravated by the fact that global change can modulate the architecture of species interaction networks (Tylianakis, Didham, Bascompte, & Wardle, 2008). The transformation of natural habitats into arable land and the introduction of invasive species changes species composition drastically and in a short time, requiring permanent adaptations to new interaction partners. Species loss in one trophic level may cause secondary species extinctions in the other level, thereby reducing network robustness, that is, the capacity of a network to buffer such secondary extinctions (Memmott, Waser, & Price, 2004). Generalization and nestedness may generally increase network robustness, because species have alternative interaction partners, suggesting changing sensitivity of networks to species loss along environmental gradients.

It is assumed that specialization in plant–pollinator networks is linked to functional traits, which restrict species flexibility to switch between different interaction partners (Dehling, Jordano, Schaefer, Böhning‐Gaese, & Schleuning, 2016; Stang, Klinkhamer, Waser, Stang, & Meijden, 2009). Broad‐scale correlations between specialization and species traits provide important information about such trait‐based feedback on specialization, but have hardly been studied on a community level in insects (Albrecht et al., 2018; Lara‐Romero et al., 2019). Bees and syrphid flies, for example, differ in their requirements for floral resources. In bees—but not in syrphid flies—the whole offspring depends on the pollen selection (Praz, Müller, & Dorn, 2008), which might increase bees' selectiveness. Similarly, morphological traits like, for example, proboscis length could restrict the number of potential interaction partners (Ibanez, 2012). Physiological and energetic constraints are suggested to shape the mean and the variance of species traits along elevational gradients (Classen, Steffan‐Dewenter, Kindeketa, & Peters, 2017; Hoiss, Krauss, Potts, Roberts, & Steffan‐Dewenter, 2012), indicating that morphological barriers restricting the choice of interaction partners can change with increasing elevation.

Here, we analyzed patterns of specialization of plants and pollinators at different levels of organization in natural and disturbed habitats along a 3.4 km elevational gradient on Mt. Kilimanjaro (Tanzania, East Africa). First, we tested the hypothesis that the structure of plant–pollinator networks changes along elevational gradients. In particular, we hypothesized that species and plant–pollinator networks are more specialized in the warm lowlands than in the cool highlands, as energy restrictions should favor generalization in higher elevations. Second, we aimed to explain changes in network structure by a set of major factors that are assumed to have a positive effect on specialization. Using path analysis, we separated the direct and indirect effects of resource availability, pollinator richness and visitation rates, temperature, and habitat area on the community mean of pollinator specialization. Third, we tested whether changes in the specialization of pollinator species are related to taxonomic identity, morphological traits or species elevational ranges. Finally, we explored whether changes in the architecture of plant–pollinator networks lead to a higher sensitivity of some elevational zones to simulated species extinctions.

## 2 MATERIALS AND METHODS

### 2.1 Study sites

We selected 19 100 × 100 m study sites on the southern slopes of Mt. Kilimanjaro, spanning an elevational gradient from 993 m above sea level (m a.s.l.) up to 4,390 m a.s.l. Study sites covered the major natural and anthropogenic habitat types of Mt. Kilimanjaro: colline savanna and maize fields (990–1,020 m a.s.l.), lower montane forest, agroforestry systems (Chagga home gardens), grasslands, coffee plantations (1,260–1,920 m a.s.l.), montane undisturbed and disturbed by former logging *Ocotea* forest (2,120–2,470 m a.s.l.), upper montane undisturbed and fire‐disturbed *Podocarpus* forest (2,850–2,990 m a.s.l.), subalpine *Erica* forest (3,880 m a.s.l.), and alpine *Helichrysum* vegetation (3,880–4,390 m a.s.l.; Table S1). The average distance between study sites was 22.6 ± *SD* 13.1 km; only two sites were nearer than 2 km (1,920 m), which is still above the average foraging ranges of most pollinators. Along this elevational gradient mean annual temperature varies between 3.1 and 24.0°C, and mean annual precipitation ranges between 590 and 2,740 mm with maximal precipitation around 2,200 m a.s.l. (Hemp, 2006; Figure S1.3).

### 2.2 Plant–pollinator interactions, species identification, and pollinator traits

In total, we conducted 80 four‐hour transect walks on the selected sites, summing up to 320 observational hours. Transect walks were conducted over the course of two consecutive years (2011, 2012), covering different seasons of the year. We recorded plant–pollinator interactions between 07.30 and 17.00 hr on days when the weather was sunny or moderately cloudy. In case of rain, mist or heavy wind, we interrupted transect walks and continued it as soon as the weather was suitable again—but at the latest within the next 2 days. Due to logistic constraints and often unsuitable climatic conditions like rain or dense fog at high elevations, the number of transect walks was not homogeneously distributed among sites but ranged between one and eight (Table S1.1). We addressed this by using networks deriving from individual transect walks as sampling units within a mixed‐effects model framework (see Section 2.5). This approach ensures that all species contributing to one network co‐occurred in space and time. Additionally, it reduces the susceptibility of network metrics to errors in species identification, because morphospecies were separated only within but not across networks. During each transect walk, we moved slowly and without fixed corridors through the vegetation of each site and recorded each interaction in which a pollinator touched reproductive parts of herbaceous plant species or bushes. If pollinators visited different flowers of the same plant individual before catching, we counted it as single interaction. Note that flower visitors are termed “pollinators” here, although their contribution to the pollination success is unknown. In 95% of all considered interactions, we either identified pollinator species in the field (*Apis mellifera*), or caught them with sweep nets for further identification by experienced taxonomists. Escaped pollinators, that is, 5% of considered interactions, were also recorded and separated with a conservative approach within single networks (see Appendix S1 for detailed information). Exemplars of interacting plant species, including both herbs and shrubs, were collected or photographed and identified by the botanist AH on a species level. Nomenclature follows the Flora of Tropical East Africa (FTEA, 1952–2012).

We restricted our analyses to pollinator taxa, which we could sort on a species or morphospecies level (46% and 54% of all specimens, respectively). Most major groups of pollinators were included, that is, all Hymenoptera: Apoidea: Apiformes (“bees”), the paraphyletic group of nonbee aculeates and symphyta (“wasps”), and Diptera: Syrphidae (“syrphid flies”). Butterflies were excluded from analyses because only relatively few interactions were observed, and the voucher sampling success of the few specimens was poor. In addition, we excluded nonsyrphid Diptera from analyses, because reliable species delimitation based on outer morphology was not feasible for this group (see also Table S1.4). We further restricted analyses to networks with a minimum of five interactions, but for most networks we could sample a much higher number of interactions (mean number of observed interactions ± *SD* = 64.9 ± 69.4). This filtering resulted in 67 networks and led to the exclusion of one network collected in the disturbed *Ocotea* forest, thereby reducing the number of sites from 19 to 18.

We measured pollinator's proboscis length and head widths using a stereo microscope with calibrated ocular micrometer and a precision of 0.01 mm. Trait matching between proboscis length and corolla tube lengths might restrict the number of interaction partners and has been linked to species specialization before (Miller‐Struttmann et al., 2015). Head width, used as a proxy for body size (Branquart & Hemptinne, 2000), is related to energy requirements and foraging ranges (Greenleaf, Williams, Winfree, & Kremen, 2007) and can thus be related to species specialization. To calculate trait means, we measured the traits of all available, but not more than 10 individuals per species and study site (three individual measurements for syrphid flies) and averaged those values per species. We assessed elevational ranges of pollinator species by subtracting the minimum from the maximum elevation of occurrence. See Appendix S1 for more details on trait measurements and range estimations.

### 2.3 Resources, pollinator richness, climate, area, and land use intensity

After each transect walk, we counted total flower abundance and flower richness within 10 4 × 5 m rectangles and used the sum of all flower heads and the total number of species as estimates for flower abundance and flower richness per transect walk. Replicated pan trap sampling across seasons was used to estimate network‐independent species richness of pollinator species per site (Figure S1.2a). Pan traps were installed in the same years when transect walks were conducted. Sampling effort was equal on all study sites here. Species richness of the pan trap sampling was correlated with the total species richness of pollinators per site collected with nets (Pearson correlation, *r* = .7, *p* < .001). Visitation rates were calculated by dividing the number of observed interactions per transect walk (as a measure for insect activity) by flower abundance. Temperature was recorded on each site using temperature loggers (Appelhans et al., 2016). We calculated two temperature measures: For the mean annual temperature (MAT), we averaged all measurements per study site. For the mean temperature during each transect walk (ACT = “actual temperature”), we averaged all measurements during each 4‐hr transect walk. Mean annual precipitation (MAP) was interpolated for every study site using a kriging approach on the basis of 15‐year‐long data records from a network of about 70 rain gauges on Mount Kilimanjaro (Appelhans et al., 2016).

Study sites differed in their land use intensity. To account for that, we used a quantitative composite index of human land use, hereafter termed LUI, which was designed in earlier studies based on data collected on 60 study sites (Peters et al., 2019). In a nutshell, we averaged standardized estimates of (a) annual plant biomass removal and (b) agricultural inputs (irrigation, fertilization, insecticides, fungicide, and herbicides) and quantified (c) differences of the vegetation structure to the natural vegetation (quantified in terms of canopy closure, canopy height, vegetation heterogeneity) on each study site. Changes in structural characteristics with elevation are partly natural and independent of land use intensification (e.g., different canopy cover in savannah and forest) such that raw data of vegetation structure would not be an informative indicator of land use. Therefore, we calculated the mean Euclidian dissimilarity of vegetation structure measures of the respective study site to the average vegetation structure in natural habitats at the same elevational level. We further quantified (d) landscape composition 1.5 km around each study site—that is, the proportion of areas with agriculturally managed habitats. We standardized all components (a–d), before averaging them to the final index. More details on this index and other variables (resources, pollinator richness, and temperature) are given in Appendix S1 and Peters et al. (2019).

Elevational belt area was extracted from a digital elevation model of Mt. Kilimanjaro with a resolution of 30 m and an extension from 37.00074 to 37.75602 E and 3.507533 to 2.750183 S (Appendix S1: Figure S1.2b). We calculated the area within a range of 100 m below and 100 m above the respective study site.

### 2.4 Network indices

We used the R package “bipartite” (Dormann, 2011; Dormann, Gruber, & Fründ, 2008) to calculate matrix size, dependence (or “interaction strength”) asymmetry and nestedness for all networks. For nestedness, we chose the “weighted nestedness overlap and decreasing fills” metric for quantitative networks (Almeida‐Neto & Ulrich, 2011). Matrix size equals the product of the number of plant and pollinator species included in each network. Dependence asymmetry ranges between −1 and 1; positive values indicate higher dependences in the higher trophic level (Bascompte, 2006; Blüthgen et al., 2007). High values of nestedness indicate high tendencies of specialists to interact with generalists, which tend to interact among each other, while values around zero indicate the opposite. As absolute values of nestedness partly depend on network size, we standardized them by comparing observed values with results of null models (Dormann, Fründ, Blüthgen, & Gruber, 2009; Appendix S2).

We quantified the degree of specialization at different levels of biological organization: species specialization was calculated for each pollinator and plant species by the d' index, also implemented in the R package “bipartite.” The d' index ranges between zero and one, indicating maximal generalization and maximal specialization, respectively. It describes to which extent the observed interaction frequencies of plant and pollinator species deviate from expected frequencies based on random pattern of interactions considering the total frequency of interactions of each partner available (Blüthgen, Fründ, Vázquez, & Menzel, 2008). Compared with alternative specialization indices, d' is relatively robust to observation effort, that is, the specialization of rarely observed species is not overestimated (Poisot, Canard, Mouquet, & Hochberg, 2012).

We averaged d' both on a community and on a species level. The community mean of d' is the average specialization of all plants/pollinators contributing to one network (i.e., all interactions sampled during one transect walk), whereas the species mean d' is the average specialization of single pollinator species across different sites (see Appendix S2 for details). As also the dispersion of d' in a community might be of ecological relevance, we extracted the standard deviation of d' in communities and divided it by the respective community mean (=coefficient of variance [CV]).

For entire networks, we calculated Blüthgens' network specialization index *H*_2_′ using the *H2fun* function implemented in the “bipartite” package (Blüthgen et al., 2006; Dormann et al., 2009). *H*_2_′ describes complementary specialization on a network level and has been shown to be robust against sampling intensity and network size, making it a useful tool for the comparison of networks across multiple habitats. Five networks had to be excluded from analyses because we recorded only one pollinator or one plant species (three cases) during the 4‐hr walks, or because the distribution of interactions in the network matrix did not allow the generation of more than one random (shuffled) network, as required for the calculation of *H*_2_′ (two cases). To confirm that specialization patterns (*d*′ and *H*_2_′) are not driven by network properties like the observed number of interactions or matrix size, we additionally compared those indices with indices derived from null models (Table S2.2a).

Finally, we estimated network robustness against pollinator and plant extinctions for each network using the *robustness* function of the “bipartite” package. Network robustness equals the area below a secondary extinction curve. This was derived from the stepwise, random removal of species from one trophic level—by setting all entries of this species to zero—, and counting the number of secondarily extinct species from the other trophic level, that is, species with no interactions remaining. As also network robustness may partly depend on network size, we again standardized this metric through comparisons with null models, as described for nestedness in Appendix S2.

### 2.5 Statistical analyses

We analyzed how (log‐transformed) matrix size, dependence asymmetry, nestedness, *H*_2_′, community mean of *d*′ of pollinators and plants and their CV, as well as standardized network robustness (against simulated pollinator and plant extinction) changed along the elevation gradient. We fitted linear mixed‐effects (lme) models with elevation as single predictor variable and added study site as a random term, to control for repeated measurements on the same sites. *R* ^2^ values were obtained using the Nakagawa & Schielzeth approach (Nakagawa & Schielzeth, 2013) implemented in the R package “r2glmm” (Jaeger, 2016). We conducted several sensitivity analyses to test the stability of detected patterns: (a) As LUI may significantly influence patterns of biotic variables along the elevation gradient (Peters et al., 2019), and because LUI is correlated to elevation (Pearson's *r* = −.57; Table S1.3), we examined if LUI is potentially a better predictor of network metrics than elevation. We did this by comparing models with elevation as a predictor variable with models that included LUI as single predictor variable using the Akaike information criterion (AIC; Table 1). Since the sample size was low compared with the number of estimated parameters, we employed the AIC_C_ with a second‐order bias correction quantifying model support. (b) Network metrics calculated from single sampling days might be strongly influenced by actual weather conditions and might not be representative of the “average” interaction patterns. To verify the robustness of our results in this respect, we additionally calculated the mean of each metric per study site and analyzed the elevational pattern with ordinary linear models (*N* = 18; Table S2.2b). (c) We further tested whether the comparatively poorly sampled high‐elevated habitats with generally low interaction rates are responsible for the strong elevational patterns detected in specialization, by restricting analyses to all study sites below 2,000 m a.s.l. (d) To illuminate whether the differences in the number of repeated measurements per site influences the elevational pattern of *H*_2_′, and to test if higher sampling effort per study site leads to different patterns of network metrics along the elevation gradient, we lumped a minimum of five replicated transect walks per site to one joint network (possible for 10 sites, Table S1.1), extracted *H*_2_′ and analyzed the pattern along elevation with an ordinary linear model (*N* = 10). (e) To evaluate the impact of changes in vegetation structure on elevational patterns, we analyzed the elevational trends in network and species specialization for a subset of all study sites in which the canopy cover is smaller or equal to the median canopy cover (i.e., all “open” habitats).

**Table 1:** Outputs of linear mixed‐effects models, showing the changes in network metrics along the elevational gradient on Mt. Kilimanjaro

Network metric	Predictor	*N*	*G*	*SD* rand.	Estimate	*SE*	*df*	*t*‐Value	*p*‐Value	*R* ^2^	ΔAICc
log (matrix size)	Intercept	67	18	.69	5.12	.42	49	12.28			
	Elevation				−8.3E–04	1.7E–04	16	−4.86	<.001	.37	−8.74
Dependence asymmetry	Intercept	67	18	.10	.25	.08	49	3.16			
	Elevation				−9.24E–05	3.675E–05	16	−2.52	.023	.11	0.99
Std. nestedness	Intercept	61	16	.52	−4.17	.56	45	−7.45			
	Elevation				8.9E–04	2.9E–04	14	3.08	.008	.15	2.09
Mean *d*′ pollinators	Intercept	66	17	.09	.64	.05	49	11.98			
	Elevation				−1.5E–04	2.3E–05	15	−6.60	<.001	.53	13.11
Mean *d*′ plants	Intercept	66	17	.16	.79	.09	49	8.67			
	Elevation				−1.7E–04	3.8E–05	15	−4.57	<.001	.36	8.60
*H*_2_′	Intercept	62	17	.11	.89	.08	45	10.88			
	Elevation				−1.9E–04	3.9E–05	15	−4.77	<.001	.32	7.34
Std. robustness (against pollinator extinction)	Intercept	64	17	.54	−3.06	.66	47	−4.67			
	Elevation				7.6E–04	3.4E–04	15	2.23	.041	.08	2.91
Std. robustness (against plant extinction)	Intercept	64	17	.56	−4.30	.60	47	−7.16			
	Elevation				1.0E–03	3.1E–04	15	3.28	.005	.17	2.24

Note

All models were fitted with elevation as fixed factor and study site as a random term. ΔAIC_C_ gives AIC_C_ differences of the presented model to a model that includes LUI as single fixed factor. A negative ΔAIC_C_ indicates that the LUI model performed better than the model with elevation; |ΔAIC_C_| ≤ 2 indicates, that the two models were similarly supported by the data.

Abbreviations: *df*, degrees of freedom; *G*, number of study sites; *N*, number of networks included in analysis; *R* ^2^, semipartial *R* ^2^ for the fixed effect; *SE*, standard error.

We conducted path analyses to separate the direct and indirect effects of flower abundance, flower richness, pollinator richness, visitation rates, temperature, and habitat area on the community mean of pollinator specialization, *d*′. We based the a priori structure of the path model on the following hypotheses (Figure 2a). (a) Pollinator specialization is predicted to increase with flower abundance and/or the number of flowering plant species (*=resource‐driven specialization*), as these factors determine whether specialization is feasible in a habitat, which needs to provide sufficient amount of food for pollinators. MAT, MAP, and LUI will influence flower abundance and flower richness. (b) Pollinator specialization is predicted to increase with the frequency of interactions among pollinators (=*pollinator‐driven specialization)*. Specialization might be a strategy to avoid competition among pollinators (Brosi & Briggs, 2013). Interaction frequencies will increase either with pollinator richness and/or with visitation rates, that is the ratio between the abundance or activity of pollinators and the number of resources. Pollinator richness will depend on MAT, MAP, LUI, area and mean richness and abundance of flowers. Per calculation, visitation rates depend on insect activity and flower abundances, but should further be influenced by ACT, which increases the activity of ectothermic pollinators. (c) Temperature is predicted to have a direct, positive impact on the mean specialization of pollinators (=*temperature‐driven specialization*). ACT will directly control the costs of an observed foraging flight. In contrast, MAT reflects the temperature that is experienced by communities on a long term and is probably correlated with the number of hours per day suitable for foraging. MAT might further positively correlate with evolutionary rates and with this the likelihood that specialists evolve (Allen et al., 2006; Lin et al., 2019). (d) Pollinator specialization is expected to increase with increasing habitat area (=*area‐driven specialization*), as specialists typically depend on larger areas than generalists do.

All variables included in the path model were standardized by *z*‐transformation using the *scale* function in R. To avoid correlation between exogenous variables and to improve the ratio between the number of model parameter and the number of observations, we selected variables within and across hypotheses via model selection based on AIC_C_ prior to path analysis (Table S2.3). After variable selection, all possible path combinations were tested and compared by the AIC_C_ of the respective path model. We used Fisher's C as a goodness of fit parameter. Statistical details on the preselection process and on path analyses are given in Appendix S2.3.

Intraspecific variation of pollinator specialization along the temperature gradient was analyzed for pollinators that occurred on at least three different sites (43 species). We used generalized linear mixed‐effects models with Gaussian error distribution, MAT as single fixed factor and species and site as crossed random effects (Bates, Mächler, Bolker, & Walker, 2015). As this analysis might be especially error‐prone toward species misidentification, we run the same model also on a subdataset that only included species with proper species names (20 species).

Differences in pollinator species specialization of different pollinator groups, that is, Hymenoptera (bees and wasps) versus Diptera (syrphid flies), as well as the association of species d' with proboscis length, head width, and elevational range size were investigated across all pollinators and within pollinator groups with linear mixed‐effects models. We partly controlled for nonindependence between species by integrating taxonomic information as nested random terms in the models, that is, “genus” for differences between pollinator groups, and “order/family/genus” for differences between species traits. We restricted trait analyses to pollinators that we observed at least three times (*n* = 87).

We tested if network robustness against plant or pollinator extinction is influenced by *H*_2_′, standardized nestedness, MAT and LUI, which were included as additive explaining variables using lme models. Study site was added as random term here again to account for the hierarchical structure of the data. For model simplification, nonsignificant terms (*p* > .05) were successively removed from the model.

## 3 RESULTS

In total, we analyzed 4,380 plant–pollinator interactions (3,757 bee, 196 wasp, and 427 hoverfly interactions). We identified 141 plant species (123 species, two species complexes, 16 morphospecies), and 187 pollinator species (84 species, 103 morphospecies). More details on the taxonomic resolution are given in the Appendices S1 (Table S1.4) and S3.

### 3.1 Elevational patterns in plant–pollinator networks

Networks (*N* = 67) included between 1 and 32 pollinator species and between 1 and 14 plant species. The relative proportions of bees, hoverflies, and wasps in the pollinating community did not significantly change along the elevational gradient (*p* > .05). Matrix size ranged between 2 and 448 (mean: 78.9 ± 85.4), and significantly decreased along the elevational gradient (Table 1). Dependence asymmetry declined with elevation: In the lowlands, positive values of dependence asymmetry indicated higher dependencies of pollinators on plants, while from an elevation of about 2,730 m a.s.l. these dependencies, on average, turned around. Nestedness increased with elevation (Table 1). LUI, which was negatively correlated with elevation (*r* = −.57), was in case of one network metric a better predictor variable than elevation (Table 1): Matrix size increased with increasing LUI (*t*‐value: 6.949, *p* < .001, *R* ^2^ = .61).

**Figure 1:**
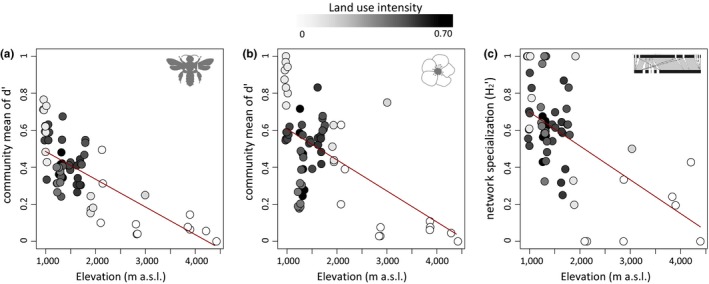
Change of plant–pollinator specialization along the elevational gradient on Mt. Kilimanjaro at species and network level. (a) Community mean of pollinator specialization (*d*'), (b) community mean of plant specialization (*d*') and (c) plant‐pollinator network specialization (*H*_2_′) decreased with increasing elevation (m a.s.l. = meters above sea level). Dots represent the abundance‐weighted means of species specialization indices (*d*′) and the *H*_2_′ values per transect walk. Lines represent predicted relationships derived from linear mixed‐effects models with elevation as single predictor variable and site as a random term. Dot colors indicate the strength of land use intensity

The community mean of pollinator and plant specialization (*d*′), as well as network specialization (*H*_2_′) decreased with elevation (Figure 1a–c, Table 1). Comparisons with null models indicated that these declines of specialization are not driven by network size or interaction frequencies alone (Table S2.2a). Also, sensitivity analyses confirmed the robustness of the majority of reported patterns: First, all elevational patterns in network metrics were also statistically significant, when analyzing metric means per study site with linear models (*N* = 18; Table S2.2b). Second, significant declines of network and mean pollinator (but not plant) specialization with elevation were not only depending on the inclusion of high elevation sites but also detectable for the more intensively sampled study sites below 2,000 m a.s.l. (*p* < .05). Third, the decline in H_2_' was also detected when five networks per sites were lumped to one joint network, indicating that the pattern was robust to differences in sampling effort (lm, *N* = 10, *t* = −2.631, *p* < .03). Finally, the decline of specialization in networks, as well as in pollinator and plant communities, was stable when analyzing only sites with low canopy cover (≤median canopy cover), indicating that vegetation structure did not influence the results (all *p* < .05).

While the *d*′ of pollinators on average decreased with elevation the coefficient of variation (CV) of pollinator d' increased (pollinators: *t* = 4.76, *p* < .001, *R* ^2^ = .27), indicating more variability in the level of specialization in higher elevations. However, this trend was not detectable for study sites below 2,000 m a.s.l. (*p* > .05). The CV in the plant d' did not significantly change with elevation (*t* = 1.59, *p* = .135).

Interestingly, a decline in pollinator specialization was also detected at the intraspecific level as the level of specialization of pollinator species decreased along the temperature gradient, that is, species that showed specialized foraging behavior in warm areas, tended to forage more generally in cooler regions (lmer, *df* = 39.76, *t* = 4.746, *p* < .001). This trend was also found when restricting analysis to species with proper species name (lmer, *df* = 57.95, *t* = 2.76, *p* = .008). However, the proportion of explained variance was low in both cases (*R* ^2^ = .13 and *R* ^2^ = .09, respectively).

### 3.2 Drivers of specialization in pollinator communities

**Figure 2:**
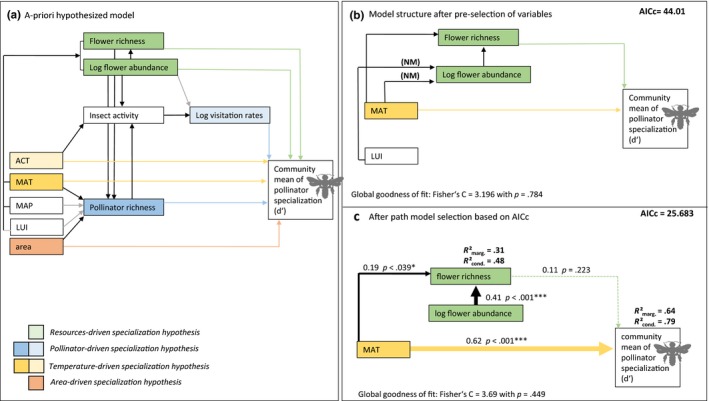
Direct and indirect predictors of mean pollinator specialization on Mt. Kilimanjaro. (a) A priori hypothesized causal structure of the model. Competitive variables within each hypothesis were highlighted with similar colors. Black and colored arrows indicate positive relationship expectations, gray arrows negative relationships. (b) Structure of the full path model after semiautomated preselection of variables. Detailed information on the preselection process are given in the method section. (c) Final path model derived by AIC_C_‐based model selection across all possible paths combinations presented in b. Path coefficients and related *p*‐Values, as well as both marginal and conditional *R* ^2^ values for all response variables are presented. Dashed lines indicate nonsignificant paths. The presented path model is statistically not distinguishable from a model in which flower richness has no impact on pollinator specialization (ΔAIC_C_ = 0.05). The global goodness of fit of all path models was estimated with Fisher's C. *p*‐values > .05 for C indicate that the specific causal structure reflects the data properly. ACT, actual temperature; LUI, land use intensity, MAP, mean annual precipitation; MAT, mean annual temperature; area = habitat area (100 m above and 100 m below the respective study site). All variables were *z*‐transformed prior to analyses. Statistical details are given in Table S2.3

Mean annual temperature, pollinator richness, flower richness, and habitat area were significantly related to the drop in the community mean of pollinator specialization, when analyzing these variables in separate linear mixed‐effects models (Table S2.3). When we added these variables in a joint mixed‐effects model and evaluated the support for the full model and all nested models, models including only MAT or MAT and flower richness received the highest level of support (Table S2.3). The best path model revealed that MAT was the strongest predictor of the community mean of pollinator specialization (cond. *R* ^2^ = 79%; Figure 2c). MAT had both a direct positive effect on pollinator specialization, and an indirect positive effect via flower richness, which was weak compared to the direct effect (Figure 2c). In the best path model, (log‐transformed) flower abundance had a positive effect on flower richness. We tested a set of competing alternative path combinations and ranked path models according to their AIC_C_ values. A path model that did not include the link from flower richness to pollinator specialization was statistically not distinguishable from the presented model (ΔAIC_C_ = 0.05).

### 3.3 Pollinator specialization and species traits

**Figure 3:**
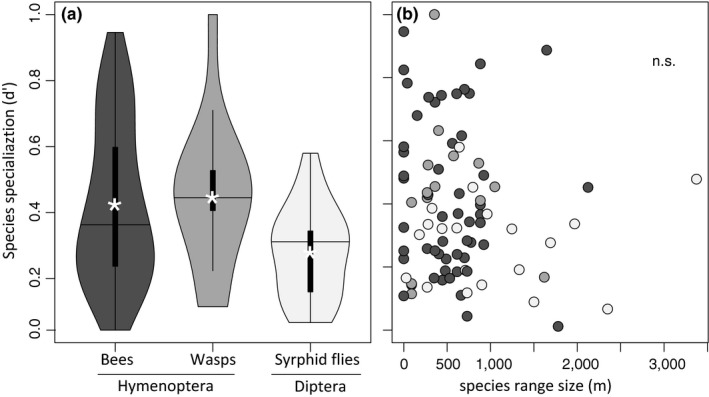
Impact of taxonomy and species elevational range on pollinator species specialization (*d*′). (a) Hymenoptera (bees and wasps) were on average more specialized than Diptera (syrphid flies; *t* = 2.70, *p* = .010; bees‐ wasps: *t* = 0.03, *p* = .74; bees—syrphid flies: *t* = −2.52, *p* = .016; syrphid flies—wasps: *t* = 3.36, *p* = .002). While black box plots present common summary statistics (with data medians as black line and means as white asterisks), the surrounding violin plots signal (smoothed) probability density of the data at different values. (b) Pollinator specialization was not related to the elevational range size of pollinator species. Dot color in (b) corresponds to the different taxonomic groups, as introduced in (a). We considered only pollinators that we observed at least three times (87 species)

Pollinator specialization (*d*′) was generally higher in Hymenoptera than in Diptera (lme, *t* = 2.312, *p* = .043, Figure 3a), but was neither related to species elevational range sizes (Figure 3b), nor to functional traits (glossa lengths: lme, *t* = 0.406, *p* = .687; head width: lme, *t* = 0.305, *p* = .762).

### 3.4 Robustness

**Figure 4:**
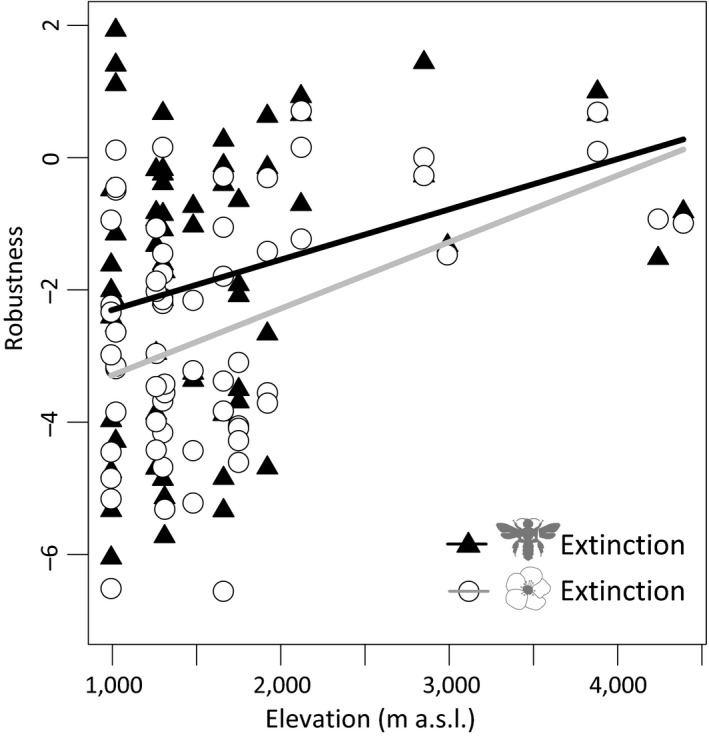
Network robustness against species extinction along the elevational gradient on Mt. Kilimanjaro. Robustness against pollinator extinctions (black triangles) exceeded robustness against plant extinction (white dots); both metrics increased with elevation. Robustness was standardized via null‐model comparison prior to analysis. Lines represent predicted relationships derived from linear mixed‐effects models with elevation as single predictor variable and site as a random term

Network robustness against plant extinction was on average lower than network robustness against pollinator extinction (lme, *t* = −2.70, *p* = .008), and both increased with elevation (Figure 4, Table 1). Among MAT, LUI, *H*_2_′, and nestedness, network specialization (*H*_2_′) was the best predictor of network robustness, with less specialized networks being more robust (lme, *n* = 62, *df* = 44, against plant extinction: *t* = −3.48, *p* < .001, *R* ^2^ = .23; against pollinator extinction: *t* = −2.94, *p* = .004, *R* ^2^ = .14).

## 4 DISCUSSION

### 4.1 Elevational patterns in plant–pollinator networks

Plant–pollinator networks showed clear broad‐scale patterns along the elevational gradient of Mt. Kilimanjaro, highlighting the importance of ecological and/or evolutionary drivers in structuring species interactions. Specifically, we found an increase in generalization with elevation on the species (*d*′ intraspecific), community (community mean of *d*′), and network level (*H*_2_′). Similar patterns in the specialization of plant–insect pollinator interactions have been reported from biogeographically independent regions, including the Alps (Hoiss et al., 2015, network level), Colorado Rocky Mountains (Miller‐Struttmann & Galen, 2014, intraspecific and network level), the Andes (Ramos‐Jiliberto et al., 2010, network level), and an island volcano in Tenerife, Canary Islands (Lara‐Romero et al., 2019, network and community level). Far less consistent are the patterns reported from plant–hummingbird interactions along elevational gradients, which were either more generalized in the highlands (Maglianesi, Blüthgen, Böhning‐Gaese, & Schleuning, 2015), or in the lowlands (Dalsgaard et al., 2018). Similarly contradicting are the results from meta‐analyses describing specialization patterns along other temperature gradients, that is, latitudinal gradients. Here, patterns ranged from higher pollinator specialization in higher (Schleuning et al., 2012), or lower latitudes respectively (Trøjelsgaard & Olesen, 2013), to no trend at all (Ollerton & Cranmer, 2002). In view of the fact that temperature has a big impact on specialization, we suggest that besides the difficulty to standardize studies properly with different sampling strategies for latitudinal meta‐analyses, the dominance of endothermic pollinators (e.g., hummingbirds, bats) toward the tropics might partly explain these discrepancies. For example, in hummingbirds species richness, contemporary precipitation and quaternary climate change velocity, but not MAT, predicted specialization (Dalsgaard et al., 2011). Depending on the ratio of endothermic and ectothermic pollinators considered in meta‐analyses, the role of temperature in structuring specialization patterns might become weaker or stronger causing different patterns along latitude.

In our study, we concentrated on three important insect pollinator groups: bees, nonbee aculeates (“wasps”), and hoverflies. With this we excluded some flower visitors, which are known to contribute to pollination like nonsyrphid Diptera (Orford, Vaughan, & Memmott, 2015). These Diptera were more abundant in higher than in lower elevations abundant in high than in low elevations. However, as they are known to be as specialized as syrphid flies (Orford et al., 2015), and, in accordance with syrphid flies, less specialized than Hymenoptera (Benadi, Hovestadt, Poethke, & Blüthgen, 2014, and our results), the exclusion of this group in our analyses unlikely affected the general direction of the reported patterns of specialization.

### 4.2 Drivers of specialization

Along the slopes of Mt. Kilimanjaro, MAT was the best predictor of mean pollinator specialization. Even within pollinator species, individuals tended to forage more generalized in the cold highlands than in the warm lowlands. The mechanisms behind the link of temperature and specialization remain elusive. However, one major hypothesis is that is the increase of metabolic costs in foraging flights under cool temperatures (Kovac et al., 2015; Stabentheiner, Vollmann, Kovac, & Crailsheim, 2003), triggers generalized foraging. Even within pollinator species, individuals tended to forage more generalized in the highlands than in the lowlands, supporting the concept of energetic constraints. Long‐tongued bumble bees species also showed comparable shifts in intraspecific foraging behavior in alpine habitats (Miller‐Struttmann & Galen, 2014). The authors suggested that restricted seasonal lengths drive generalized resource usage of bumble bees in cold habitats, which is plausible in temperate, but not in cold tropical mountain habitats that lack distinct seasonality. We suggest that in tropical highlands energy‐limitations are caused directly by temperature, as temperature controls the foraging costs for ectothermic pollinators (Kovac et al., 2015; Stabentheiner et al., 2003). Furthermore, in the alpine and subalpine zone, we observed that foraging of bees was restricted to very few warm and cloud‐free hours of a day, while in the lowlands bee foraging took place all day long. Alternatively or in parallel, higher evolutionary rates under warm climates might favor the evolution of specialists. For instance, bumble bee species within the same subgenera have been shown to evolve faster in low elevations than in the highlands (Lin et al., 2019). This parallels with higher bee richness in the lowlands (Classen et al., 2015; Hoiss et al., 2012) and a higher chance for the evolution of true specialists. In addition, we detected a potential indirect effect of MAT on specialization via flower richness. Flower resource diversity, and a sufficient amount of flowers per resource type, might be a premise for pollinator specialization, as specialized pollinators need to acquire enough food.

Against expectations and in contrast to other studies (Dalsgaard et al., 2011; Schleuning et al., 2012), pollinator species richness and visitation rates were poor predictors of pollinator specialization. This may indicate that physiological and energetic constraints set by temperature rather than the interactions among pollinators (e.g., competitive interactions), shape insect pollinator specialization at Mt. Kilimanjaro. This is also reflected by a previously reported decline of intraspecific variation in morphological traits of wild bees with increasing elevation (Classen et al., 2017): if reduced competition was the major force shaping these functional traits of bees in high elevations, the opposite pattern, that is, “character release,” would be expected.

Despite the high proportion of explained variance in pollinator specialization in the path model (*R* ^2^ cond. = .79), and even though we verified our results by a set of robustness analyses, it must be considered that our study is based on correlation analyses and that the power of statistical tools to separate variables that are correlated in nature (e.g., area and MAT; pollinator richness and MAT), are restricted. We also cannot rule out that factors correlating with temperature, which we could not consider in this study, affected specialization. For example, it is conceivable that pollinator generalization in the highlands coincides with the dominance of plant species with open flowers and short nectar‐holding tubes like, for example, *Helichrysum* species, which are accessible by more or other, more generalized, pollinator species than tubular flowers. Additional, more detailed analyses of plant–pollinator interactions and their traits in the field in combination with true experiments will be necessary to verify the results of our correlative study.

### 4.3 Species traits

We showed that Hymenoptera were on average more specialized than Diptera, which is in agreement with reports from the literature (Benadi et al., 2014; Weiner, Werner, Linsenmair, & Blüthgen, 2011) and which can be explained by the fact that bee larval survival depends on the pollen source (Praz et al., 2008). Morphological traits were not related to the degree of species specialization, indicating that traits of one trophic level are not informative to predict specialization (Dalsgaard et al., 2018). Trait matching between trophic levels, in contrast, can sharpen our understanding about how species traits structure species interactions and network architecture (Albrecht et al., 2018; Bender et al., 2018; Dehling et al., 2016). Interestingly, we found no link between species specialization and species elevational ranges. Thus, specialized foraging does not necessarily restrict pollinators' ability to inhabit new habitats, which is in line with our finding that even within species the degree of specialization can be adapted to changing abiotic conditions.

### 4.4 Robustness

Network robustness against pollinator extinction was generally higher than robustness against plant extinction. This is in line with simulation studies under climate change scenarios (Schleuning et al., 2016). Importantly, network robustness increased with elevation. In other words, network sensitivity to species loss was highest where habitat transformation and land use intensification, as main driving forces of species loss are most prevalent (Nogués‐Bravo, Araújo, Romdal, & Rahbek, 2008). Robustness against species extinction was negatively correlated with network specialization, but not with nestedness, as shown in other studies (Bastolla et al., 2009; Rohr, Saavedra, & Bascompte, 2014). Network generalization decreases the co‐dependence of interaction partners and is thus an important driver of network robustness. We assessed robustness against random species extinctions of one trophic level, as we currently had no concrete indication for other extinction orders than random. Especially in human‐modified landscapes, abundant and well‐connected species are frequent visitors of crops, suffering from pesticide usage, soil cultivation or the bee‐keeping associated spread of pathogens. They might nowadays face extinction risks that are comparable with the ones of less‐abundant and more specified species. Alternatively, trait‐based extinction orders can drastically alter the robustness pattern along elevational gradients and related pollination services in unpredictable ways (Larsen, Williams, & Kremen, 2005). Generally, the robustness metric needs to be handled with care. First, species may adapt to a certain degree to other interaction partners, when their previous partner goes extinct. Our finding that specified species from the lowlands tended to forage more generalized in the highlands, points toward such an adaptive capacity, which will relax the co‐dependence of certain species and strengthen network robustness (Kaiser‐Bunbury, Muff, Memmott, Müller, & Caflisch, 2010). Second, robustness was calculated for networks that derived from short‐term observations. However, many pollinators show floral fidelity, making observed networks more specialized and thus less robust than networks observed over a longer period (Petanidou et al., 2008; Spiesman & Gratton, 2016). Finally, this approach ignores that the loss of one species can change the behavior or another species from the same trophic level, with consequences for interaction partners and network stability (Brosi & Briggs, 2013).

## 5 CONCLUSIONS

Plant–pollinator network architecture strongly responded to changing climate along the slopes of Mt. Kilimanjaro. We identified MAT as the main driving force for specialization, which in turn affected network robustness against species extinction. Rising metabolic costs for foraging flights in cool environments might explain the detected decrease of (ectothermic) pollinator specialization in the highlands. We expect that the temperature‐dependence of metabolic costs affects also the structure of other species interactions including ectothermic organisms (e.g., parasitoid‐host, plant‐herbivores), with consequences for diversity, ecosystem functions and stability (Plowman et al., 2017).

Although the mechanisms behind the temperature—specialization relationship remain speculative, the outstanding role of temperature in structuring plant–pollinator interactions is alarming: global temperatures are predicted to increase by up to 0.7°C in the next two decades (compared to 1985–2005; IPCC, 2013). So far, climate change was expected to disrupt plant–pollinator interactions by causing spatial and phenological mismatches between interaction partners (Hegland, Nielsen, Lázaro, Bjerknes, & Totland, 2009), or by increased frequency of extreme weather events (Hoiss et al., 2015), with negative consequences for interaction resilience and fitness of plants and pollinators (Forrest, 2015; Schenk, Krauss, & Holzschuh, 2018). The direct impact of temperature on the specialization of species, which has also been reported from small‐scale climatic gradients for network specialization (Petanidou et al., 2018), imposes additional challenges to species interactions. The loss of interaction partners may improve pollination quality on small spatial and temporal scales, as less heterospecific pollen will be transported (Brosi, 2016). This, however, may drastically reduce pollination insurance in the long term (Brosi, 2016).

We conclude that rising temperatures in the course of climate change will destabilize species interactions along entire elevational gradients, thereby exerting additional pressure on species, which already live close to their maximum thermal capacity (Colwell, Brehm, Cardelus, Gilman, & Longino, 2008).

## CONFLICT OF INTEREST

None declared.

## AUTHOR CONTRIBUTIONS

ISD, MKP, and AC designed the study; AC conducted the fieldwork, compiled the data, and conducted statistical analyses with support of MKP. AH selected the study sites and identified all plant species. Insects (bees, syrphid flies, wasps) were identified by CDE, AS and RSP, respectively. AC wrote the first draft of the manuscript, while all authors contributed to the final version.

## Data Availability

Data supporting the finding of this study (species lists, plant–pollinator interactions, and pollinator traits) are made available in the supplements. Data are additionally archived in the PANGAEA database at: https://doi.org/10.1594/PANGAEA.911390.

## References

[ece36056-bib-0001] Albrecht, J. , Classen, A. , Vollstädt, M. G. R. , Mayr, A. , Mollel, N. P. , Schellenberger Costa, D. , … Schleuning, M. (2018). Plant and animal functional diversity drive mutualistic network assembly across an elevational gradient. Nature Communications, 9, 3177. 10.1038/s41467-018-05610-w PMC6085337 30093613

[ece36056-bib-0002] Allen, A. P. , Gillooly, J. F. , Savage, V. M. , & Brown, J. H. (2006). Kinetic effects of temperature on rates of genetic divergence and speciation. Proceedings of the National Academy of Sciences of the United States of America, 103, 9130–9135. 10.1073/pnas.0603587103 16754845 PMC1474011

[ece36056-bib-0003] Almeida‐Neto, M. , & Ulrich, W. (2011). A straightforward computational approach for measuring nestedness using quantitative matrices. Environmental Modelling and Software, 26, 173–178. 10.1016/j.envsoft.2010.08.003

[ece36056-bib-0004] Appelhans, T. , Mwangomo, E. , Otte, I. , Detsch, F. , Nauss, T. , & Hemp, A. (2016). Eco‐meteorological characteristics of the southern slopes of Kilimanjaro, Tanzania. International Journal of Climatology, 36, 3245–3258. 10.1002/joc.4552

[ece36056-bib-0005] Bairey, E. , Kelsic, E. D. , & Kishony, R. (2016). High‐order species interactions shape ecosystem diversity. Nature Communications, 7, 12295. 10.1038/ncomms12285 PMC4974637 27481625

[ece36056-bib-0006] Bascompte, J. , & Jordano, P. (2007). Plant‐animal mutualistic networks: The architecture of biodiversity. Annual Review of Ecology Evolution and Systematics, 38, 567–593. 10.1146/annurev.ecolsys.38.091206.095818

[ece36056-bib-0007] Bascompte, J. , Jordano, P. , & Olesen, J. M. (2006). Asymmetric coevolutionary networks facilitate biodiversity maintenance. Science, 312, 431–433. 10.1126/science.1123412 16627742

[ece36056-bib-0008] Bastolla, U. , Fortuna, M. A. , Pascual‐García, A. , Ferrera, A. , Luque, B. , & Bascompte, J. (2009). The architecture of mutualistic networks minimizes competition and increases biodiversity. Nature, 458, 1018–1020. 10.1038/nature07950 19396144

[ece36056-bib-0009] Bates, D. , Mächler, M. , Bolker, B. , & Walker, S. (2015). Fitting linear mixed‐effects models using lme4. Journal of Statistical Software, 67, 1–48.

[ece36056-bib-0010] Benadi, G. , Hovestadt, T. , Poethke, H.‐J. , & Blüthgen, N. (2014). Specialization and phenological synchrony of plant‐pollinator interactions along an altitudinal gradient. Journal of Animal Ecology, 83, 639–650. 10.1111/1365-2656.12158 24219131

[ece36056-bib-0011] Bender, I. M. A. , Kissling, W. D. , Blendinger, P. G. , Böhning‐Gaese, K. , Hensen, I. , Kühn, I. , … Schleuning, M. (2018). Morphological trait matching shapes plant‐frugivore networks across the Andes. Ecography, 41(11), 1910–1919. 10.1111/ecog.03396

[ece36056-bib-0012] Blüthgen, N. , Fründ, J. , Vázquez, D. P. , & Menzel, F. (2008). What do interaction network metrics tell us about specialization and biological traits? Ecology, 89, 3387–3399. 10.1890/07-2121.1 19137945

[ece36056-bib-0013] Blüthgen, N. , Menzel, F. , & Blüthgen, N. (2006). Measuring specialization in species interaction networks. BMC Ecology, 6, 9. 16907983 10.1186/1472-6785-6-9 PMC1570337

[ece36056-bib-0014] Blüthgen, N. , Menzel, F. , Hovestadt, T. , Fiala, B. , & Blüthgen, N. (2007). Specialization, constraints, and conflicting interests in mutualistic networks. Current Biology, 17, 341–346. 10.1016/j.cub.2006.12.039 17275300

[ece36056-bib-0015] Bommarco, R. , Biesmeijer, J. C. , Meyer, B. , Potts, S. G. , Pöyry, J. , Roberts, S. P. M. , … Öckinger, E. (2010). Dispersal capacity and diet breadth modify the response of wild bees to habitat loss. Proceedings of the Royal Society B: Biological Sciences, 277, 2075–2082. 10.1098/rspb.2009.2221 PMC2880091 20219735

[ece36056-bib-0016] Branquart, E. , & Hemptinne, J.‐L. (2000). Selectivity in the exploitation of floral resources by hoverflies (Diptera: Syrphinae). Ecography, 23, 732–742. 10.1111/j.1600-0587.2000.tb00316.x

[ece36056-bib-0017] Brosi, B. J. (2016). Pollinator specialization: From the individual to the community. New Phytologist, 210, 1190–1194. 10.1111/nph.13951 27038018

[ece36056-bib-0018] Brosi, B. J. , & Briggs, H. M. (2013). Single pollinator species losses reduce floral fidelity and plant reproductive function. Proceedings of the National Academy of Sciences of the United States of America, 110, 13044–13048. 10.1073/pnas.1307438110 23878216 PMC3740839

[ece36056-bib-0019] Carvell, C. , Jordan, W. C. , Bourke, A. F. G. , Pickles, R. , Redhead, J. W. , & Heard, M. S. (2012). Molecular and spatial analyses reveal links between colony‐specific foraging distance and landscape‐level resource availability in two bumble bee species. Oikos, 121, 734–742. 10.1111/j.1600-0706.2011.19832.x

[ece36056-bib-0020] Chan, S. , Shih, W. , Chang, A. , Shen, S. , & Chen, I. (2019). Contrasting forms of competition set elevational range limits of species. Ecology Letters, 22, 1668–1679. 10.1111/ele.13342 31347240

[ece36056-bib-0021] Chittka, L. , & Thomson, J. D. (1997). Sensori‐motor learning and its relevance for task specialization in bumble bees. Behavioral Ecology and Sociobiology, 41, 385–398. 10.1007/s002650050400

[ece36056-bib-0022] Classen, A. , Peters, M. K. , Kindeketa, W. J. , Appelhans, T. , Eardley, C. D. , Gikungu, M. W. , … Steffan‐Dewenter, I. (2015). Temperature versus resource constraints: Which factors determine bee diversity on Mount Kilimanjaro, Tanzania? Global Ecology and Biogeography, 24, 642–652. 10.1111/geb.12286

[ece36056-bib-0023] Classen, A. , Steffan‐Dewenter, I. , Kindeketa, W. J. , & Peters, M. K. (2017). Integrating intraspecific variation in community ecology unifies theories on body size shifts along climatic gradients. Functional Ecology, 31, 768–777. 10.1111/1365-2435.12786

[ece36056-bib-0024] Colwell, R. K. , Brehm, G. , Cardelus, C. L. , Gilman, A. C. , & Longino, J. T. (2008). Global warming, elevational range shifts, and lowland biotic attrition in the wet tropics. Science, 322, 258–261. 10.1126/science.1162547 18845754

[ece36056-bib-0025] Dalsgaard, B. O. , Kennedy, J. D. , Simmons, B. I. , Baquero, A. C. , Martín González, A. M. , Timmermann, A. , … Rahbek, C. (2018). Trait evolution, resource specialization and vulnerability to plant extinctions among Antillean hummingbirds. Proceedings of the Royal Society B: Biological Sciences, 285, 20172754 10.1098/rspb.2017.2754 PMC5897636 29563263

[ece36056-bib-0026] Dalsgaard, B. O. , Magård, E. , Fjeldså, J. , Martín González, A. M. , Rahbek, C. , Olesen, J. M. , … Svenning, J.‐C. (2011). Specialization in plant‐hummingbird networks is associated with species richness, contemporary precipitation and quaternary climate‐change velocity. PLoS ONE, 6, e25891 10.1371/journal.pone.0025891 21998716 PMC3187835

[ece36056-bib-0027] Dehling, D. M. , Jordano, P. , Schaefer, H. M. , Böhning‐Gaese, K. , & Schleuning, M. (2016). Morphology predicts species’ functional roles and their degree of specialization in plant–frugivore interactions. Proceedings of the Royal Society B: Biological Sciences, 283, 20152444 10.1098/rspb.2015.2444 PMC4795026 26817779

[ece36056-bib-0028] Dormann, C. F. (2011). How to be a specialist? Quantifying specialization in pollination networks. Network Biology, 1, 1–20.

[ece36056-bib-0029] Dormann, C. F. , Fründ, J. , Blüthgen, N. , & Gruber, B. (2009). Indices, graphs and null models: Analyzing bipartite ecological networks. Open Ecology Journal, 2, 7–24. 10.2174/1874213000902010007

[ece36056-bib-0030] Dormann, C. F. , Gruber, B. , & Fründ, J. (2008). Introducing the bipartite package: Analysing ecological networks. R News, 8, 8–11.

[ece36056-bib-0031] Forrest, J. R. K. (2015). Plant‐pollinator interactions and phenological change: What can we learn about climate impacts from experiments and observations? Oikos, 124, 4–13. 10.1111/oik.01386

[ece36056-bib-0032] FTEA (1952). Flora of tropcial East Africa. London, UK: FTEA.

[ece36056-bib-0033] Garibaldi, L. A. , Steffan‐Dewenter, I. , Winfree, R. , Aizen, M. A. , Bommarco, R. , Cunningham, S. A. , … Klein, A. M. (2013). Wild pollinators enhance fruit set of crops regardless of honeybee abundance. Science, 339, 1608–1611. 10.1126/science.1230200 23449997

[ece36056-bib-0034] Goulson, D. , Lye, G. C. , & Darvill, B. (2008). Diet breadth, coexistence and rarity in bumble bees. Biodiversity and Conservation, 17, 3269–3288. 10.1007/s10531-008-9428-y

[ece36056-bib-0035] Greenleaf, S. S. , Williams, N. M. , Winfree, R. , & Kremen, C. (2007). Bee foraging ranges and their relationship to body size. Oecologia, 153, 589–596. 10.1007/s00442-007-0752-9 17483965

[ece36056-bib-0036] Hegland, S. J. , Nielsen, A. , Lázaro, A. , Bjerknes, A.‐L. , & Totland, Ø. (2009). How does climate warming affect plant‐pollinator interactions? Ecology Letters, 12, 184–195. 19049509 10.1111/j.1461-0248.2008.01269.x

[ece36056-bib-0037] Hemp, A. (2006). Continuum or zonation? Altitudinal gradients in the forest vegetation of Mt. Kilimanjaro. Plant Ecology, 184, 27–42. 10.1007/s11258-005-9049-4

[ece36056-bib-0038] Hoiss, B. , Krauss, J. , Potts, S. G. , Roberts, S. , & Steffan‐Dewenter, I. (2012). Altitude acts as an environmental filter on phylogenetic composition, traits and diversity in bee communities. Proceedings of the Royal Society B: Biological Sciences, 279, 4447–4456. 10.1098/rspb.2012.1581 PMC3479805 22933374

[ece36056-bib-0039] Hoiss, B. , Krauss, J. , & Steffan‐Dewenter, I. (2015). Interactive effects of elevation, species richness and extreme climatic events on plant‐pollinator networks. Global Change Biology, 21, 4086–4097. 10.1111/gcb.12968 26332102

[ece36056-bib-0040] Ibanez, S. (2012). Optimizing size thresholds in a plant–pollinator interaction web: Towards a mechanistic understanding of ecological networks. Oecologia, 170, 233–242. 10.1007/s00442-012-2290-3 22415527

[ece36056-bib-0041] IPCC (2013). Climate change 2013: The physical science basis. Contribution of Working Group I to the Fifth Assessment Report of the International Panel on Climate change. Cambridge, UK and New York, NY: Cambridge University Press.

[ece36056-bib-0042] Jaeger, B. (2016). r2glmm: Computes R squared for mixed (multilevel) models. R package version 0.1.1. Retrieved from https://CRAN.R-project.org/package=r2glmm

[ece36056-bib-0043] Jordano, P. , Bascompte, J. , & Olesen, J. M. (2002). Invariant properties in coevolutionary networks of plant‐animal interactions: Invariant properties in coevolutionary networks. Ecology Letters, 6, 69–81. 10.1046/j.1461-0248.2003.00403.x

[ece36056-bib-0044] Kaiser‐Bunbury, C. N. , Muff, S. , Memmott, J. , Müller, C. B. , & Caflisch, A. (2010). The robustness of pollination networks to the loss of species and interactions: A quantitative approach incorporating pollinator behaviour. Ecology Letters, 13, 442–452. 10.1111/j.1461-0248.2009.01437.x 20100244

[ece36056-bib-0045] Kovac, H. , Stabentheiner, A. , & Brodschneider, R. (2015). What do foraging wasps optimize in a variable environment, energy investment or body temperature? Journal of Comparative Physiology A, 201, 1043–1052. 10.1007/s00359-015-1033-4 PMC4611018 26286881

[ece36056-bib-0046] Kunin, W. , & Iwasa, Y. (1996). Pollinator foraging strategies in mixed floral arrays: Density effects and floral constancy. Theoretical Population Biology, 49, 232–263. 10.1006/tpbi.1996.0013 8813024

[ece36056-bib-0047] Lara‐Romero, C. , Seguí, J. , Pérez‐Delgado, A. , Nogales, M. , & Traveset, A. (2019). Beta diversity and specialization in plant–pollinator networks along an elevational gradient. Journal of Biogeography, 46, 1598–1610. 10.1111/jbi.13615

[ece36056-bib-0048] Larsen, T. H. , Williams, N. M. , & Kremen, C. (2005). Extinction order and altered community structure rapidly disrupt ecosystem functioning. Ecology Letters, 8, 538–547. 10.1111/j.1461-0248.2005.00749.x 21352458

[ece36056-bib-0049] Lin, G. , Huang, Z. , Wang, L. , Chen, Z. , Zhang, T. , Gillman, L. N. , & Zhao, F. (2019). Evolutionary rates of bumble bee genomes are faster at lower elevations. Molecular Biology and Evolution, 36, 1215–1219. 10.1093/molbev/msz057 30865278 PMC6526908

[ece36056-bib-0050] Maglianesi, M. A. , Blüthgen, N. , Böhning‐Gaese, K. , & Schleuning, M. (2015). Functional structure and specialization in three tropical plant‐hummingbird interaction networks across an elevational gradient in Costa Rica. Ecography, 38, 1119–1128. 10.1111/ecog.01538

[ece36056-bib-0051] Memmott, J. , Waser, N. M. , & Price, M. V. (2004). Tolerance of pollination networks to species extinctions. Proceedings of the Royal Society B: Biological Sciences, 271, 2605–2611. 10.1098/rspb.2004.2909 PMC1691904 15615687

[ece36056-bib-0052] Miller‐Struttmann, N. E. , & Galen, C. (2014). High‐altitude multi‐taskers: Bumble bee food plant use broadens along an altitudinal productivity gradient. Oecologia, 176, 1033–1045. 10.1007/s00442-014-3066-8 25199658

[ece36056-bib-0053] Miller-Struttmann, N. E. , Geib, J. C. , Franklin, J. D. , Kevan, P. G. , Holdo, R. M. , Ebert-May, D. , … & Galen, C. (2015). Functional mismatch in a bumble beepollination mutualism under climate change. Science, 349, 1541–1544. 10.1126/science.aab0868 26404836

[ece36056-bib-0054] Morris, R. J. , Gripenberg, S. , Lewis, O. T. , & Roslin, T. (2014). Antagonistic interaction networks are structured independently of latitude and host guild. Ecology Letters, 17, 340–349. 10.1111/ele.12235 24354432 PMC4262010

[ece36056-bib-0055] Nakagawa, S. , & Schielzeth, H. (2013). A general and simple method for obtaining R2 from generalized linear mixed‐effects models. Methods in Ecology and Evolution, 4, 133–142.

[ece36056-bib-0056] Nogués‐Bravo, D. , Araújo, M. B. , Romdal, T. , & Rahbek, C. (2008). Scale effects and human impact on the elevational species richness gradients. Nature, 453, 216–219. 10.1038/nature06812 18464741

[ece36056-bib-0057] Novotny, V. (2006). Why are there so many species of herbivorous insects in tropical rainforests? Science, 313, 1115–1118. 10.1126/science.1129237 16840659

[ece36056-bib-0058] Olesen, J. M. , Bascompte, J. , Dupont, Y. L. , & Jordano, P. (2007). The modularity of pollination networks. Proceedings of the National Academy of Sciences of the United States of America, 104, 19891–19896. 10.1073/pnas.0706375104 18056808 PMC2148393

[ece36056-bib-0059] Ollerton, J. , & Cranmer, L. (2002). Latitudinal trends in plant‐pollinator interactions: Are tropical plants more specialised? Oikos, 98, 340–350. 10.1034/j.1600-0706.2002.980215.x

[ece36056-bib-0060] Orford, K. A. , Vaughan, I. P. , & Memmott, J. (2015). The forgotten flies: The importance of non‐syrphid Diptera as pollinators. Proceedings of the Royal Society B: Biological Sciences, 282, 20142934–20142934. 10.1098/rspb.2014.2934 PMC4389612 25808886

[ece36056-bib-0061] Petanidou, T. , Kallimanis, A. S. , Lazarina, M. , Tscheulin, T. , Devalez, J. , Stefanaki, A. , … Sgardelis, S. P. (2018). Climate drives plant‐pollinator interactions even along small‐scale climate gradients: The case of the Aegean. Plant Biology, 20, 176–183. 10.1111/plb.12593 28637086

[ece36056-bib-0062] Petanidou, T. , Kallimanis, A. S. , Tzanopoulos, J. , Sgardelis, S. P. , & Pantis, J. D. (2008). Long‐term observation of a pollination network: Fluctuation in species and interactions, relative invariance of network structure and implications for estimates of specialization: High plasticity in plant‐pollinator networks. Ecology Letters, 11, 564–575. 10.1111/j.1461-0248.2008.01170.x 18363716

[ece36056-bib-0063] Peters, M. K. , Hemp, A. , Appelhans, T. , Becker, J. N. , Behler, C. , Classen, A. , … Steffan‐Dewenter, I. (2019). Climate–land‐use interactions shape tropical mountain biodiversity and ecosystem functions. Nature, 568, 88–92. 10.1038/s41586-019-1048-z 30918402

[ece36056-bib-0064] Plowman, N. S. , Hood, A. S. C. , Moses, J. , Redmond, C. , Novotny, V. , Klimes, P. , & Fayle, T. M. (2017). Network reorganization and breakdown of an ant–plant protection mutualism with elevation. Proceedings of the Royal Society B: Biological Sciences, 284, 20162564 10.1098/rspb.2016.2564 PMC5360921 28298349

[ece36056-bib-0065] Poisot, T. , Canard, E. , Mouquet, N. , & Hochberg, M. E. (2012). A comparative study of ecological specialization estimators: Species‐level specialization. Methods in Ecology and Evolution, 3, 537–544. 10.1111/j.2041-210X.2011.00174.x

[ece36056-bib-0066] Praz, C. J. , Müller, A. , & Dorn, S. (2008). Specialized bees fails to develop on non‐host pollen: Do plants chemically protect their pollen? Ecology, 89, 795–804. 10.1890/07-0751.1 18459342

[ece36056-bib-0067] Ramos, S. E. , & Schiestl, F. P. (2019). Rapid plant evolution driven by the interaction of pollination and herbivory. Science, 364, 193–196. 10.1126/science.aav6962 30975889

[ece36056-bib-0068] Ramos‐Jiliberto, R. , Domínguez, D. , Espinoza, C. , López, G. , Valdovinos, F. S. , Bustamante, R. O. , & Medel, R. (2010). Topological change of Andean plant – Pollinator networks along an altitudinal gradient. Ecological Complexity, 7, 86–90. 10.1016/j.ecocom.2009.06.001

[ece36056-bib-0069] Rohr, R. P. , Saavedra, S. , & Bascompte, J. (2014). On the structural stability of mutualistic systems. Science, 345, 1253497–1253497. 10.1126/science.1253497 25061214

[ece36056-bib-0070] Schenk, M. , Krauss, J. , & Holzschuh, A. (2018). Desynchronizations in bee‐plant interactions cause severe fitness losses in solitary bees. Journal of Animal Ecology, 87, 139–149. 10.1111/1365-2656.12694 28502082

[ece36056-bib-0071] Schleuning, M. , Fründ, J. , Klein, A.‐M. , Abrahamczyk, S. , Alarcón, R. , Albrecht, M. , … Blüthgen, N. (2012). Specialization of mutualistic interaction networks decreases toward tropical latitudes. Current Biology, 22, 1925–1931. 10.1016/j.cub.2012.08.015 22981771

[ece36056-bib-0072] Schleuning, M. , Fründ, J. , Schweiger, O. , Welk, E. , Albrecht, J. , Albrecht, M. , … Hof, C. (2016). Ecological networks are more sensitive to plant than to animal extinction under climate change. Nature Communications, 7, 13965 10.1038/ncomms13965 PMC5196430 28008919

[ece36056-bib-0073] Song, C. , Rohr, R. P. , & Saavedra, S. (2017). Why are some plant – Pollinator networks more nested than others? Journal of Animal Ecology, 86, 1417–1424. 10.1111/1365-2656.12749 28833083

[ece36056-bib-0074] Spiesman, B. J. , & Gratton, C. (2016). Flexible foraging shapes the topology of plant‐pollinator interaction networks. Ecology, 97, 1431–1441. 10.1890/15-1735.1 27459774

[ece36056-bib-0075] Stabentheiner, A. , Vollmann, J. , Kovac, H. , & Crailsheim, K. (2003). Oxygen consumption and body temperature of active and resting honeybees. Journal of Insect Physiology, 49, 881–889. 10.1016/S0022-1910(03)00148-3 16256690

[ece36056-bib-0076] Stang, M. , Klinkhamer, P. G. L. , Waser, N. M. , Stang, I. , & van der Meijden, E. (2009). Size‐specific interaction patterns and size matching in a plant‐pollinator interaction web. Annals of Botany, 103, 1459–1469. 10.1093/aob/mcp027 19228701 PMC2701768

[ece36056-bib-0077] Sugiura, S. (2010). Species interactions‐area relationships: Biological invasions and network structure in relation to island area. Proceedings of the Royal Society B: Biological Sciences, 277, 1807–1815. 10.1098/rspb.2009.2086 PMC2871870 20147330

[ece36056-bib-0078] Trøjelsgaard, K. , & Olesen, J. M. (2013). Macroecology of pollination networks. Global Ecology and Biogeography, 22, 149–162. 10.1111/j.1466-8238.2012.00777.x

[ece36056-bib-0079] Tylianakis, J. M. , Didham, R. K. , Bascompte, J. , & Wardle, D. A. (2008). Global change and species interactions in terrestrial ecosystems. Ecology Letters, 11, 1351–1363. 10.1111/j.1461-0248.2008.01250.x 19062363

[ece36056-bib-0080] Tylianakis, J. M. , Laliberté, E. , Nielsen, A. , & Bascompte, J. (2010). Conservation of species interaction networks. Biological Conservation, 143, 2270–2279. 10.1016/j.biocon.2009.12.004

[ece36056-bib-0081] Vaudo, A. D. , Patch, H. M. , Mortensen, D. A. , Tooker, J. F. , & Grozinger, C. M. (2016). Macronutrient ratios in pollen shape bumble bee (*Bombus impatiens*) foraging strategies and floral preferences. Proceedings of the National Academy of Sciences of the United States of America, 113, E4035–E4042. 27357683 10.1073/pnas.1606101113 PMC4948365

[ece36056-bib-0082] Waser, N. M. , & Ollerton, J. (2006). Plant‐pollinator interactions. From specialization to generalization. Chicago, IL: University of Chicago Press.

[ece36056-bib-0083] Weiner, C. N. , Werner, M. , Linsenmair, K. E. , & Blüthgen, N. (2011). Land use intensity in grasslands: Changes in biodiversity, species composition and specialisation in flower visitor networks. Basic and Applied Ecology, 12, 292–299. 10.1016/j.baae.2010.08.006

[ece36056-bib-0084] Wisz, M. S. , Pottier, J. , Kissling, W. D. , Pellissier, L. , Lenoir, J. , Damgaard, C. F. , … Svenning, J.‐C. (2013). The role of biotic interactions in shaping distributions and realised assemblages of species: Implications for species distribution modelling. Biological Reviews, 88, 15–30. 10.1111/j.1469-185X.2012.00235.x 22686347 PMC3561684

