# Supplementary material for: Specialization of plant–pollinator interactions increases with temperature at Mt. Kilimanjaro
Source: Ecol Evol. 2020 Feb 5;10(4):2182–95. doi: 10.1002/ece3.6056 (PMC7042760; doi:10.1002/ece3.6056)
Supplement: Supplementary file 1 [file ECE3-10-2182-s001.pdf]

S1 Methodological details

S1.1 Study design

Table S1.1 Overview of the study sites, their characteristics and the number of transect walks (TW) per site. Study sites differed in habitat type, elevation, and land use intensity (LUI). Lower sites were located outside the National Park (NP) of Mt. Kilimanjaro, higher sites inside. Due to weather conditions and logistic constraints, replicates were not homogenously distributed along the elevational gradient. We controlled for this in all statistical analyses. We analyzed all plant-pollinator interactions including bees, wasps and syrphid flies, and restricted our analyses to networks with at least five interactions (mean number of observed interactions ± standard deviation = 64.9 ± 69.4), explaining deviances between the number of conducted and analyzed transect walks.

siteID	habitat type	elevation	NP	LUI	# conducted TW	# analyzed TW
sav4	savannah	993	outside	0.120	8	8
mas1	maize field	1020	outside	0.688	7	7
hom4	Chagga homegarden	1260	outside	0.525	7	7
cof3	coffee plantation	1300	outside	0.865	7	7
gra5	grassland	1300	outside	0.678	3	2
gra4	grassland	1310	outside	0.631	3	3
gra3	grassland	1480	outside	0.686	4	4
gra1	grassland	1660	outside	0.732	7	7
gra2	grassland	1750	outside	0.654	5	5
flm1	forest lower montane	1920	inside	0.153	6	4
foc1	*Ocotea* forest	2120	inside	0.013	5	3
fod2	disturbed *Ocotea* forest	2470	inside	0.062	1	0
fpo1	*Podocarpus* forest	2850	inside	0.003	5	3
fpd2	disturbed *Podocarpus* forest	2990	inside	0.217	1	1
fer0	*Erica* forest	3880	inside	0.006	2	1
hel1	*Helichrysum* zone	3880	inside	0.000	5	2
hel2	*Helichrysum* zone	4190	inside	0.012	1	1
hel3	*Helichrysum* zone	4240	inside	0.002	2	1
hel4	*Helichrysum* zone	4390	inside	0.000	1	1
					**80**	**67**

S1.2 Details on variables collected on each study site

Flower resources were estimated by counting flower units within ten 4 x 5 m rectangles (Fig. S1.2). We defined a flower unit as a patch of flowers that a small pollinator of about 1 cm length would exploit by walking, instead of flying (e.g. the inflorescences of Asteraceae were counted as distinct flower

units) (Carvalheiro, Barbosa, & Memmott, 2008). We counted all flowering plants within subplots, i.e. also species that were not included in plant-pollinator networks. Network-independent **pollinator richness** (bees, wasps, syrphid flies) was assessed by installing eight pan trap clusters on 50 x 50 m subplots in the center of each study site (Fig. S1.2a). Each pan trap cluster consisted of one UV-bright blue, one yellow and one white pan (Classen et al., 2015; Peters et al., 2016; Westphal et al., 2008). Six pan trap clusters were installed along two parallel 50 m transects on each study site with a minimal distance of 15 m between them. Two pan trap clusters where installed at the outer margins of the subplots. We sampled pollinators in different vegetation heights, i.e. at ~35 cm (herbal layer) and ~120 cm (shrub layer) above ground (four traps for each vegetation height). In forest sites, we installed three additional trap clusters in the canopy (up to a mid-canopy layer of 25 m). However, for all sites we used only data of eight randomly chosen trap clusters per sampling round to guarantee equal sample intensity on all study sites. Pan traps were filled with water and a drop of liquid soap to break water surface tension, and were emptied after 48 hours, pooling the colors of one cluster into one sample. We sampled pollinators on each study site for a total of three times summing up to a total of 48 pan trap days per study site (3 sampling rounds × 8 pan trap samples x 2 sampling days). We visited study sites outside the Kilimanjaro National Park twice in 2011 and once in 2012, while study sites inside the national park were visited three times in 2012. We carefully ensured that sampling rounds on each study site covered multiple seasons *(i.e. warm dry season, cold dry season, small rainy season)* so that the potential effect of seasonal changes in species communities on diversity estimates can be assumed to be similar. Traps that were destroyed (occurred only outside the national park) were replaced by pan trap samples from a fourth sampling round that was conducted in 2011. We pooled species richness data of sampling rounds per study site for all analyses.

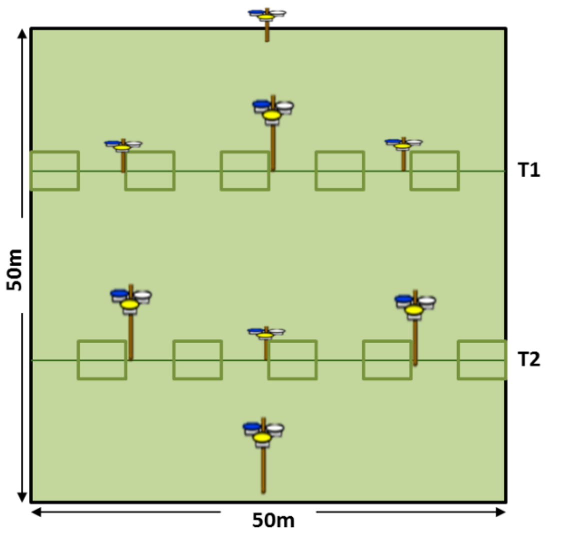

Figure S1.2a Sampling design for the variables flower abundance, flower richness and pollinator richness. Flower abundance and flower richness was counted on ten 4 x 5 m rectangles (here marked in dark green) along two 50 m transect lines (T1, T2). Pollinator richness was assessed with pan traps in different vegetation heights along the transect lines and the margin of the 50 x 50 m subplot. In case of forested plots, we installed three additional traps in the canopy (not shown here). The presented subplot was located in the center of the 100 x 100 m study site.

Temperature was recorded on each study site using temperature loggers (DK320 Humilog rugged, Driesen + Kern, Germany) that were installed 2 m above ground, covered with a plastic dish to avoid direct sunlight (Appelhans et al., 2016). Temperature was recorded for approximately 2 years at 5-min intervals. Due to various circumstances (e.g. stolen or inoperative temperature loggers), we missed temperature records for ten of the 80 transect walks. In these cases we used the average of 4 hourly predicted temperature values deriving from a linear model with all recorded temperature values of a given study site as response variable and study site, year, month and hour of the day as predictors. MAT was obtained by averaging all individual temperature measurements at a site. This value represents the temperature that species experience on average during their life cycle and to which species have probably adapted over evolutionary time scales. Mean annual temperature is therefore

supposed to filter species traits and to determine community composition. ACT was obtained by averaging all temperature values recorded during transect walks. MAT and ACT may vary substantially, even within single sites: for example while MAT at one alpine *Helichrysum* site approximated 4 °C, ACT ranged up to 12 °C. As ACT influences the energetic costs of a foraging flight, it may change pollinator behavior.

Land use was quantified via a composite index of human land use, which considered the percentage of biomass removal, the amount of agricultural inputs, the modification of vegetation structure, and the percentage of agriculture at the landscape scale (Peters et al., 2019). All measurements were conducted on 50 x 50 m sites, which lied in the center of our study sites (100 x 100 m). Annual removal of plant biomass was calculated by averaging standardized estimates of plant biomass removal on study sites. This included estimates of biomass removal by mowing, cattle grazing, ploughing, fire events, logging and firewood collection. With the exception of ploughing which was estimated on an ordinal scale (no ploughing, ploughing by hand, ploughing with a tractor), all estimates were calculated in percentage of the standing biomass removed per year. All estimates depended on repeated visits to each of the sites (> 20 times per site), and by crosschecking personal estimates with information on activities and land-use information provided by local landowners. All estimates were conducted by the same person (AH), who knows the study region since more than 15 years (Hemp, 2005, 2006a, 2006b). Agricultural inputs were calculated by averaging standardized estimates of irrigation, fertilization, insecticide, fungicide, and herbicide treatments. Estimates were done on an ordinal scale (no or very low input, medium input, high input), depending on information given by local landowners and personal observations of AH. For describing the vegetation structure, we measured the canopy closure, canopy height and vegetation heterogeneity. These measurements were taken at nine points per site, which were equally dispersed over the 50 x 50 m site, and averaged. Canopy cover was measured as the mean percentage of closed cells from four spherical canopy densiometer readings taken from the nine points in the four cardinal directions. Canopy height was measured with a laser rangefinder as maximum canopy height above ground. Vegetation heterogeneity was defined as the Shannon-Wiener

diversity of canopy cover values estimated by an observer in the field at height levels of 1, 2, 4, 8, 16, 32, and 64 m. As the natural vegetation strongly changes along elevational gradients, we could not use the raw data as a measure of land use. Raw canopy closure would be a good measure of land use, e.g., in sub montane sites where the natural vegetation, forest, has a canopy closure of 100% and managed ecosystems values of < 100 %. However, it is not suitable for ecosystems in the lowland savannah zone were even the natural vegetation has a canopy closure of ~ 10%. Therefore, we calculated the mean Euclidian dissimilarity of vegetation structure measures of the respective study site to the average vegetation structure that was measured for study sites holding natural vegetation for which, by definition, the dissimilarity to natural vegetation was assumed to be zero.

As it is well established that land use intensification at the landscape scale influences ecosystem properties at a local scale, we included a variable describing the landscape composition, i.e. the proportion of areas with managed habitats in the surrounding landscape (radius = 1500 m), as a fourth indicator of human land use. The calculation of the relative proportion of natural versus managed habitats was based on a land-cover classification of the Kilimanjaro region (unpublished) that identified 27 different habitat types (18 natural and 9 managed habitat types) in the study area. Briefly, this product is based on a maximum likelihood classification applied to four almost cloud-free Terra-ASTER scenes from 11/02/2005, 02/11/2008, 28/02/2011, and 24/02/2013. All four components of the land use index were standardized before averaging them to a final composite index of human land use. All standardizations were done by calculating the difference of individual measures to the mean of all measures divided by the range of all measures $(y_i - \bar{y})/(\max(y) - \min(y))$, producing land use intensity values of between 0 (lowest land use) and 1 (highest land use).

Importantly, a land use index that is based on all four components (biomass removal, chemical inputs, vegetation structure, landscape composition) was highly correlated with a land use index that is solely based on empirically assessed data (vegetation structure, landscape composition) ($r = 0.95$, $p < 0.001$).

Elevational belt (habitat) area

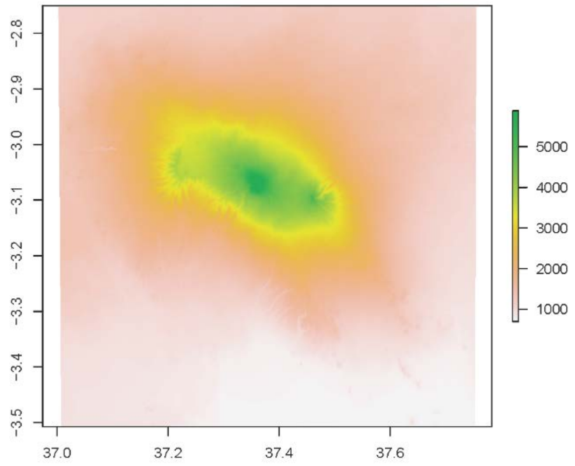

Figure S1.2b Digital elevation model from which we extracted area estimates. The DEM was derived from digitized topographical maps designed by J. A. Onginjo, C. Lambrecht and A. Hemp.

S1.3 Elevational distribution of MAT, ACT, MAP, area and LUI and correlations among variables

MAT and ACT declined linearly with elevation, while area showed an exponential decline along the elevation gradient. MAP showed a hump-shaped pattern. LUI also decreased on average along the elevational gradient, but strongly varied among habitats in lower elevations (Fig. S1.3). Simple correlation analyses revealed rather moderate to low correlation coefficients ($r \leq 0.7$, (Dormann et al., 2013). However, as expected, MAT was strongly correlated to the second temperature variable ACT ($r = 0.88$). Furthermore, MAT and area were moderately strong correlated ($r = 0.75$). As strong correlations among variables (Tab. S1.3) generally aggravates the detectability of the true drivers of patterns in e.g. pollinator specialization (Fig. 2), we tried to reduce the impact of confounding factors by pre-selecting variables before path analysis based on AIC_C (S2.3).

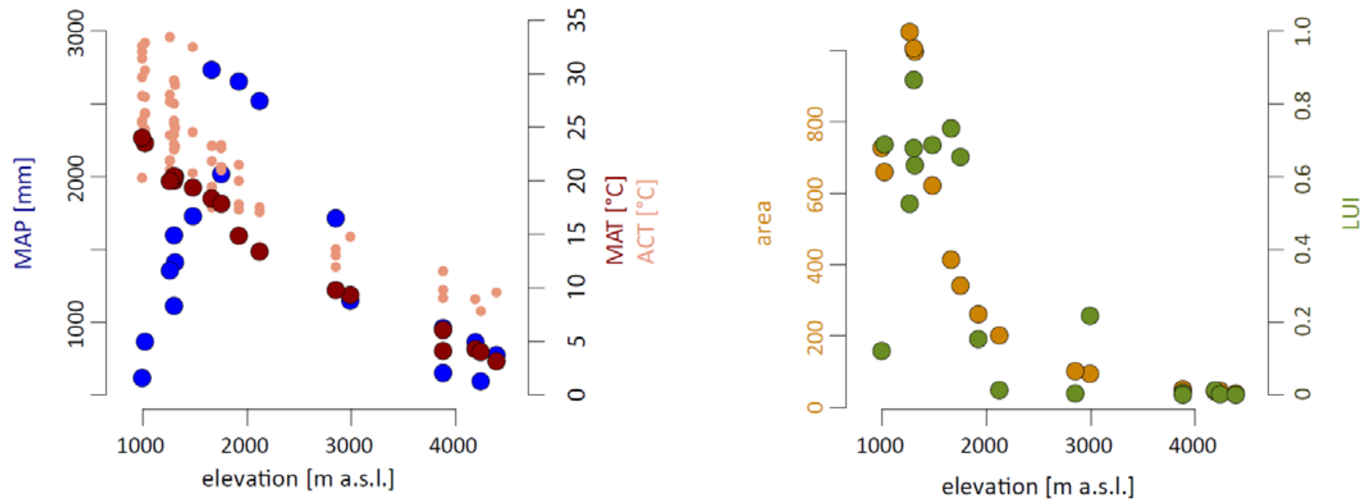

Figure S1.3 Elevational distribution of all exogenous variables included in the a-priori-hypothesized (path) model (Fig. 2a). MAP = mean annual precipitation (blue circles), MAT = mean annual temperature (dark red circles), ACT = actual temperature during the transect walk (pink circles), area = elevational belt area 100 m above and 100 m below each study site (browns circles), LUI = land use index (green circles).

Table S1.3 Pearson correlations coefficients of all exogenous variables used in the a-priori hypothesized (path) model, and elevation. All variables were z-transformed. Note that the dataset underlying the correlation matrix equals the dataset used in the path analysis; i.e. all variables are given per network. ele = elevation, ACT = actual temperature during the transect walk, MAT = mean annual temperature, MAP = mean annual precipitation, LUI = land use index, area = elevational belt area. Due to partly strong correlations, we pre-selected variables prior to path analysis (Table S2.3).

	**ele**	**ACT**	**MAT**	**MAP**	**LUI**	**area**
**ele**		-0.86	-0.98	0.00	-0.57	-0.77
**ACT**			0.88	-0.21	0.48	0.72
**MAT**				-0.14	0.55	0.75
**MAP**					0.23	-0.27
**LUI**						0.59
**area**						

S1.4 Details on species identification and trait measurements

Table S1.4 Overview about the taxonomic resolution of considered and non-considered pollinator groups. Species richness in bold, abundances in brackets.

**Group**	**#species level**	**#morphospecies level (genus based)**	**#morphospecies level (family based)**	**#individuals sorted in the field (observations)**	**#Non-considered individuals**
bees	**41** [2948]	**62** [654]	-	[155]	-
"wasps"	-	-	**38** [162]	[34]	-
hoverflies	**42** [348]	**3** [19]	**1** [15]	[45]	-
beetles	-	-	-	-	[314]
non-syrphid flies	-	-	-	-	[687]
butterflies	-	-	-	-	[295]

Species lists are presented in Appendix S3.

Classification of observed pollinators (Appendix S3, caught=0): In 234 of 4380 analysed interactions, pollinator species escaped and could thus not be sorted by taxonomists. Those species were recorded

in the field as follows: when we observed e.g. a Carpenter bee on a flower of *Tinnea aethiopica,* we named it “Xylocopa Tinnea1” in the respective dataset. When we observed a Carpenter bee that looked very different on the same plant species again, we called it “Xylocopa Tinnea2”. Similarly, when we observed undoubtedly the same pollinator species on two different plant species, we chose a common name for this pollinator. In case of doubt, we named the pollinator differently and according to the genus of the plant. This is a rather conservative approach, which might slightly overestimate the number of pollinator species; however, the exclusion of those species definitely underestimates the number of species and interactions in a network. Additionally, elimination of these species could overestimate the relative abundance of well-known species (like *Apis mellifera*), which we did not always catch when we observed it. Importantly, these observational data were excluded from all analyses, which strongly rely on proper species identification (e.g. intraspecific trend of d', range estimations, trait analyses).

Species traits: We defined head width as lateral axis of a pollinator’s head, i.e. the distance between the outermost points of the head from a frontal view – as proposed for bees (Michener, 2007). Following Harder (1982), we measured the functional unit of the proboscis, i.e. the glossa (without prementum), from the basioglossal sclerite to the distal point of the flabellum in bees and wasps. The proboscis length of syrphid flies was measured in extended position, from head to labellum tip (Ssymank, 1991) To estimate **elevational range size** of pollinator species more precisely, we supplemented species occurrence information with data from replicated pan trap sampling from another study (Classen et al., 2015; Peters et al., 2016). In this study, we assessed pollinator diversity across 60 sites distributed along the southern slopes of Mt. Kilimanjaro. We therewith sampled pollinators 127 m below the lowest and 160 m above the highest site on which we conducted transect walks, reducing the risk of sampling based underestimation of range sizes.

References cited in Supplements S1

Appelhans, T., Mwangomo, E., Otte, I., Detsch, F., Nauss, T., & Hemp, A. (2016). Eco-meteorological characteristics of the southern slopes of Kilimanjaro, Tanzania. *International Journal of Climatology*, 36(9), 3245–3258.

Carvalheiro, L. G., Barbosa, E. R. M., & Memmott, J. (2008). Pollinator networks, alien species and the conservation of rare plants: *Trinia glauca* as a case study. *Journal of Applied Ecology*, *45*(5), 1419–1427.

Classen, A., Peters, M. K., Kindeketa, W. J., Appelhans, T., Eardley, C. D., Gikungu, M. W., … Steffan-Dewenter, I. (2015). Temperature versus resource constraints: which factors determine bee diversity on Mount Kilimanjaro, Tanzania? *Global Ecology and Biogeography*, 24(6), 642–652.

Dormann, C. F., Elith, J., Bacher, S., Buchmann, C., Carl, G., Carré, G., … Lautenbach, S. (2013). Collinearity: A review of methods to deal with it and a simulation study evaluating their performance. *Ecography*, *36*(1), 27–46.

Hemp, A. (2005). Climate change-driven forest fires marginalize the impact of ice cap wasting on Kilimanjaro. *Global Change Biology*, *11*(7), 1013–1023.

Hemp, A. (2006a). Continuum or zonation? Altitudinal gradients in the forest vegetation of Mt. Kilimanjaro. *Plant Ecology*, *184*, 27–42.

Hemp, A. (2006b). The Banana Forests of Kilimanjaro: Biodiversity and Conservation of the Chagga Homegardens. *Biodiversity and Conservation*, *15*(4), 1193–1217.

Harder, L. D. (1982). Measurement and estimation of functional proboscis length in bumblebees (Hymenoptera: Apidae). *Canadian Journal of Zoology*, *60*(5), 1073–1079.

Michener, C. D. (2007). *The Bees of the World* (2nd ed.). Baltimore: The Johns Hopkins University Press.

Peters, M. K., Hemp, A., Appelhans, T., Behler, C., Classen, A., Detsch, F., … Steffan-Dewenter, I. (2016). Predictors of elevational biodiversity gradients change from single taxa to the multi-taxa community level. *Nature Communications*, *7*, 13736.

Peters, M. K., Hemp, A., Appelhans, T., Becker, J. N., Behler, C., Classen, A., … Steffan-Dewenter, I. (2019). Climate–land-use interactions shape tropical mountain biodiversity and ecosystem functions. *Nature*, *568*(7750), 88–92.

Ssymank, A. (1991). Rüssel- und Körperlänge von Schwebfliegen (Diptera: Syrphidae) unter Berücksichtigung der Verwendung von Alkoholmaterial. *Mitteilungen der Schweizerischen Entomologischen Gesellschaft*, 64, 67–80.

Westphal, C., Bommarco, R., Carré, G., Lamborn, E., Morison, N., Petanidou, T., … Steffan-Dewenter, I. (2008). Measuring bee diversity in different european habitats and biogeographical regions. *Ecological Monographs*, 78(4), 653–671.
