# Supplementary material for: Specialization of plant–pollinator interactions increases with temperature at Mt. Kilimanjaro
Source: Ecol Evol. 2020 Feb 5;10(4):2182–95. doi: 10.1002/ece3.6056 (PMC7042760; doi:10.1002/ece3.6056)
Supplement: Supplementary file 2 [file ECE3-10-2182-s002.pdf]

S2 Statistical details

S2.1 Null model for nestedness

To calculate null models we applied the Patefield algorithm, which fixes the marginal sums of the network matrix, while shuffling species interactions (1000 random matrices per network) and standardized nestedness according to the formular $z = (nest_{obs} - mean\,(nest_{rand})) / \sigma\,(nest_{rand})$.

Six networks had to be excluded from this analyses, as networks contained either only one pollinator or plant species respectively, or because nestedness of null models equaled zero (i.e. $\sigma\,(nest_{rand}) = 0$) (3 cases each).

S2.2 Calculation of d' for communities and species

We first determined d' for each species per network. To obtain one d' value per species and site, we averaged species d' within sites, weighted by the number of observed interactions of the respective species per network on given sites (Benadi, Hovestadt, Poethke, & Blüthgen, 2014). These site-based values per species were used for the analyses of **intraspecific-shifts of d'** along the elevational gradient. Furthermore, values were included in the following calculations:

Community mean of d' (community = all pollinator or plant species co-occurring within the same network): We calculated a species-weighted community mean of d' per network (i.e. each species d' contributes equally to the mean), and an abundance-weighted community mean of d' per network (i.e. d' of abundant species shape the mean more than d' of rare species). As both types of means performed very similar in analyses we here only present the abundance-weighted community means of d' (Fig. 1a, b).

Species mean of d': We averaged d' values for each pollinator species across all sites in order to link d' of pollinator species to taxonomic groups (Fig. 3a), species range sizes (Fig. 3b), and to morphological traits.

Comparison of network specialization patterns with null models: The presented specialization indices (H_2' and community mean of d') are generally quite robust against network sizes. Nevertheless, in

poorly sampled network specialization can be overestimated (Blüthgen, Menzel, & Blüthgen, 2006). Therefore, we used a null model comparison to exclude that specialization patterns are partly driven by differences in network size. For each observed network, we created 1000 random networks with the Patefield algorithm (see S2.1), and calculated mean and standard deviations of H_2' across all iterations. We then computed standardized z-scores (std. z = ($H_2'_{obs}$ – mean ($H_2'_{rand}$)) / sd ($H_2'_{rand}$)), and non-standardized z-scores (non-std. z = $H_2'_{obs}$ – mean ($H_2'_{rand}$)) (latter following Schleuning et al., 2012) for each network and analyzed those z-scores with linear mixed effects models (fixed factor: elevation, random term: study site).

Similarly, we calculated mean and standard deviation of d' for all plant and pollinator species within each network and computed standardized (std. z = (d'_{obs}- d'_{rand}) / sd (d'_{rand})) and non-standardized z-scores (non-std. z = d'_{obs}- d'_{rand}) for each species per network. We followed the protocol of S2.2, to calculate the abundance-weighted mean of these z-scores across species communities within one network and fitted those values with linear mixed effects models (fixed factor: elevation, random term: study site).

Z-scores showed the same patterns with elevation (Tab. S2.2a) as observed specialization indices, indicating that the observed specialization trends were not driven by sampling effort or network size.

Table S2.2a Output of linear mixed effects models, analyzing specialization indices on a network (H_2') and on a species level (d') (averaged for the community) after correcting for random- or network-size dependent effects via null-models. Standardized z-values follow the scheme (X_{obs}- X_{null}) / sd (X_{null}); Non-standardized z-values follow X_{obs}- X_{null}.

Response variable	predictor	Estimate	Std. Error	d.f.	t-value	p-value
std. z-value ((H2' obs - H2' rand) / sd (H2')						
	intercept	11.57	2.362	45	4.899	0
	**elevation**	-0.003	0.001	15	-2.803	0.013
non-std. z-value (H2' obs - H2' rand)						
	intercept	0.704	0.082	45	8.541	0
	**elevation**	-1.70E-04	3.93E-05	15	-4.331	< 0.001
std. z-value based community mean of d' (pollinators)						
	intercept	13.147	3.776	47	3.482	0.001
	**elevation**	-0.004	0.002	15	-2.318	0.035
non-std. z-value based community mean of d' (pollinators)						
	intercept	0.492	0.052	47	9.525	0
	**elevation**	-1.32E-04	2.40E-05	15	-5.499	< 0.001
std. z-value based community mean of d' (plants)						
	intercept	8.308	2.08	47	3.995	< 0.001
	**elevation**	-0.002	8.47E-04	15	-2.486	0.025
non-std. z-value based community mean of d' (plants)						
	intercept	0.560	0.06	47	9.318	0
	**elevation**	-1.40E-04	2.68E-05	15	-5.27	< 0.001

Table S2.2b Output of ordinary linear models, analyzing mean network indices on a study site level (N = 18).

Response variable	predictor	Estimate	Std. Error	t-value	p-value
mean matrix size (log)	intercept	5.231	0.425	12.313	
	**elevation**	-0.001	0.000	-5.203	**< 0.001**
mean dependence asymmetry	intercept	0.283	0.110	2.565	
	**elevation**	0.000	0.000	-2.571	**0.021**
mean std. nestedness	intercept	-3.986	0.450	-8.857	
	**elevation**	0.001	0.000	4.624	**< 0.001**
mean d' (pollinators)	intercept	0.633	0.053	11.921	
	**elevation**	0.000	0.000	-7.011	**< 0.001**
mean d' (plants)	intercept	0.786	0.098	7.984	
	**elevation**	0.000	0.000	-4.381	**< 0.001**
mean H2'	intercept	0.846	0.098	8.675	
	**elevation**	0.000	0.000	-4.295	**< 0.001**
mean robustness (ag. poll. ex)	intercept	-2.850	0.660	-4.319	
	**elevation**	0.001	0.000	2.598	**0.020**
mean robustness (ag. plant ex)	intercept	-4.183	0.574	-7.289	
	**elevation**	0.001	0.000	4.303	**< 0.001**

S2.3 Path analysis

Path analysis is a powerful tool to disentangle the direct and indirect factors that shape pollinator specialization along elevational gradients. However, strong correlations between exogenous variables and low sample / parameter ratios drastically reduce the reliability of this method. To avoid collinearity and to increase the sample / parameter ratio of the path model, we carefully pre-selected predictor variables:

First, we selected predictor variables of our final response (community mean of d') within hypotheses, resulting in four separate linear mixed effects models (Tab. S2.3 a-d). To account for multiple measurements on the same study site, we included study site as a random term. We evaluated the quality of all potential models by applying the dredge function implemented in the R package *MuMIn*

(Barton, 2019). This function ranks models by the AIC-based model weight. We used the AIC with a second-order bias correction (AIC_c) to account for low sample sizes compared to the number of estimated parameters. Predictor variables that were not included in the sets of best-supported models ($\Delta AIC_c \leq 1.5$) were discarded from following analyses. Note that for the selection process, we used a lower delta AIC_c value (1.5), than normally proposed in literature (e.g. AIC ≤ 2 (Burnham & Anderson, 2002)) to reduce the number of model parameters more effectively. In a second step, we selected predictor variables of the community mean of d' across hypotheses (Tab. S2.3, e), i.e. we included all predictor variables selected in the first step in one linear mixed effect model and, again, removed all predictor variables that were not included in the set of best supported models. In a third step, we used linear mixed effects models to select the predictor variables of all remaining endogenous variables ($AIC_c \leq 1.5$). Note that after pre-selection the number of predictor variables was drastically reduced and that the correlation between the remaining exogenous variables in the final path model was low (Tab. S1.3). We tested all possible path combinations of pre-selected variables (Figure 2b), using the R package *piecewiseSEM* (Lefcheck, 2016), which allows the analyses of data with a hierarchical structure. We extracted the AIC_c for each path model and selected the one with the lowest AIC_c as the best model, and alternative path models that are competitive ($\Delta AIC_c \leq 2$) (Fig. 2c). Path coefficients and p-values, as well as marginal and conditional R^2 values were obtained for each path of the best model using the summary of the *psem* function. As a goodness-of-fit test, we calculated Fisher's C, which derives from Shipley's test of directed separation. Fisher's C tests the assumption that there are no missing relationships among unconnected variables (Shipley, 2000). A Fisher´s C with a p-value > 0.05 indicates that the relationships are consistent with the data (Lefcheck, 2016).

Table S2.3 Pre-selection of variables predicting the community mean of pollinator specialization (d') for subsequent path analysis (Fig. 2). The left column shows the output of the full linear mixed effect model, the right column shows the support for the full and/ or the nested models. Most supported models (delta $AIC_c \leq 1.5$) are printed in black. All variables were z-transformed prior to analyses. Information on the selection process are given in the text above (S2.3). FRi = flower richness, FAbu = (log-transformed) flower abundance, VR = (log-transformed) visitation rates, PR = pollinator richness, MAT = mean annual temperature, ACT = actual temperature during transect walk, area = habitat area 100 m below and 100 m above the study site, Std dev (rand) = standard deviation of random effect.

Model outputs

	Value	SE	d.f.	t-value	p-value
**(a) Resource-driven specialization hypothesis**					
Intercept	-0.37	0.25	46	-1.51	0.137
flower richness	0.16	0.12	46	1.37	0.178
flower abundance (log)	-0.07	0.09	46	-0.80	0.428
Std dev (rand)	0.95				
**(b) Pollinator-driven specialization hypothesis**					
Intercept	0.16	0.22	47	0.72	0.477
visitation rates (log)	-0.04	0.07	47	-0.60	0.554
pollinator richness	0.73	0.17	15	4.18	**< 0.001**
Std dev (rand)	0.67				
**(c) Temperature-driven specialization hypothesis**					
Intercept	0.21	0.13	47	1.66	0.103
MAT	0.66	0.12	15	5.64	**< 0.001**
ACT	-0.05	0.12	47	-0.39	0.701
Std dev (rand)	0.36				
**(d) Area-driven specialization hypothesis**					
Intercept	0.04	0.21	49	0.21	0.835
area	0.62	0.15	15	4.10	**< 0.001**
Std dev (rand)	0.68				
**(e) Variable selection across hypotheses**					
Intercept	0.23	0.12	47	1.83	0.074
flower richness	0.11	0.09	47	1.22	0.229
pollinator richness	0.01	0.19	13	0.03	0.974
MAT	0.70	0.14	13	4.94	**< 0.001**
area	-0.13	0.17	13	-0.75	0.468
Std dev (rand)	0.33				

Model selection table

(a)

Int	FRi	FAbu	d.f.	logLik	AICc	delta	weight
+			3	-64.38	135.2	0.00	0.46
+	+		4	-63.76	136.2	1.03	0.275
+		+	4	-64.38	137.4	2.27	0.148
+	+	+	5	-63.43	137.9	2.73	0.118

(b)

Int	VR	PR	d.f.	logLik	AICc	delta	weight
+		+	4	-58.04	124.8	0.00	0.725
+	+	+	5	-57.86	126.7	1.98	0.269
+			3	-64.38	135.2	10.41	0.004
+	+		4	-64.33	137.3	12.57	0.001

(c)

Int	MAT	ACT	d.f.	logLik	AICc	delta	weight
+	+		4	-50.01	108.7	0.00	0.75
+	+	+	5	-49.93	110.9	2.19	0.25
+		+	4	-62.64	133.9	25.25	0.00
+			3	-64.38	135.1	26.46	0.00

(d)

Int	area	d.f.	logLik	AICc	delta	weight
+	+	4	-58.39	125.4	0.00	0.994
+		3	-64.64	135.7	10.23	0.006

(e)

Int	FRi	PR	MAT	area	d.f.	logLik	AICc	delta	weight
+			+		4	-49.88	108.4	0.00	0.317
+	+		+		5	-49.08	109.2	0.75	0.217
+			+	+	5	-49.54	110.1	1.67	0.137
+		+	+		5	-49.82	110.7	2.22	0.104
+	+		+	+	6	-48.75	111.0	2.52	0.09

References cited in Supplements S2

Barton, K. (2019). MuMIn: Multi-Model Inference. R package version 1.43.6. https://CRAN.R-project.org/package=MuMIn

Benadi, G., Hovestadt, T., Poethke, H.-J., & Blüthgen, N. (2014). Specialization and phenological synchrony of plant-pollinator interactions along an altitudinal gradient. *Journal of Animal Ecology*, *83*(3), 639–650.

Blüthgen, N., Menzel, F., & Blüthgen, N. (2006). Measuring specialization in species interaction networks. *BMC Ecology*, *6*(9).

Burnham, K. P., & Anderson, D. R. (2002). *Model Selection and Multimodel Inference: A practical information-theoretic approach* (2nd edition.). New York: Springer-Verlag.

Lefcheck, J. S. (2016). PIECEWISESEM: Piecewise structural equation modelling in R for ecology, evolution, and systematics. *Methods in Ecology and Evolution*, *7*(5), 573–579.

Schleuning, M., Fründ, J., Klein, A.-M., Abrahamczyk, S., Alarcón, R., Albrecht, M., … Blüthgen, N. (2012). Specialization of Mutualistic Interaction Networks Decreases toward Tropical Latitudes. *Current Biology*, *22*(20), 1925–1931.

Shipley, B. (2000). A New Inferential Test for Path Models Based on Directed Acyclic Graphs. *Structural Equation Modeling: A Multidisciplinary Journal*, *7*(2), 206–218.
